# Supplementary material for: Co-transcriptional Loading of RNA Export Factors Shapes the Human Transcriptome
Source: Mol Cell. 2019 Jul 25;75(2):310–323.e8. doi: 10.1016/j.molcel.2019.04.034 (PMC6675937; doi:10.1016/j.molcel.2019.04.034)
Supplement: Document S1. Figures S1–S6 and Table S1 [file mmc1.pdf]

Molecular Cell, Volume *75*

Supplemental Information

Co-transcriptional Loading of RNA Export Factors Shapes the Human Transcriptome

Nicolas Viphakone, Ian Sudbery, Llywelyn Griffith, Catherine G. Heath, David Sims, and Stuart A. Wilson

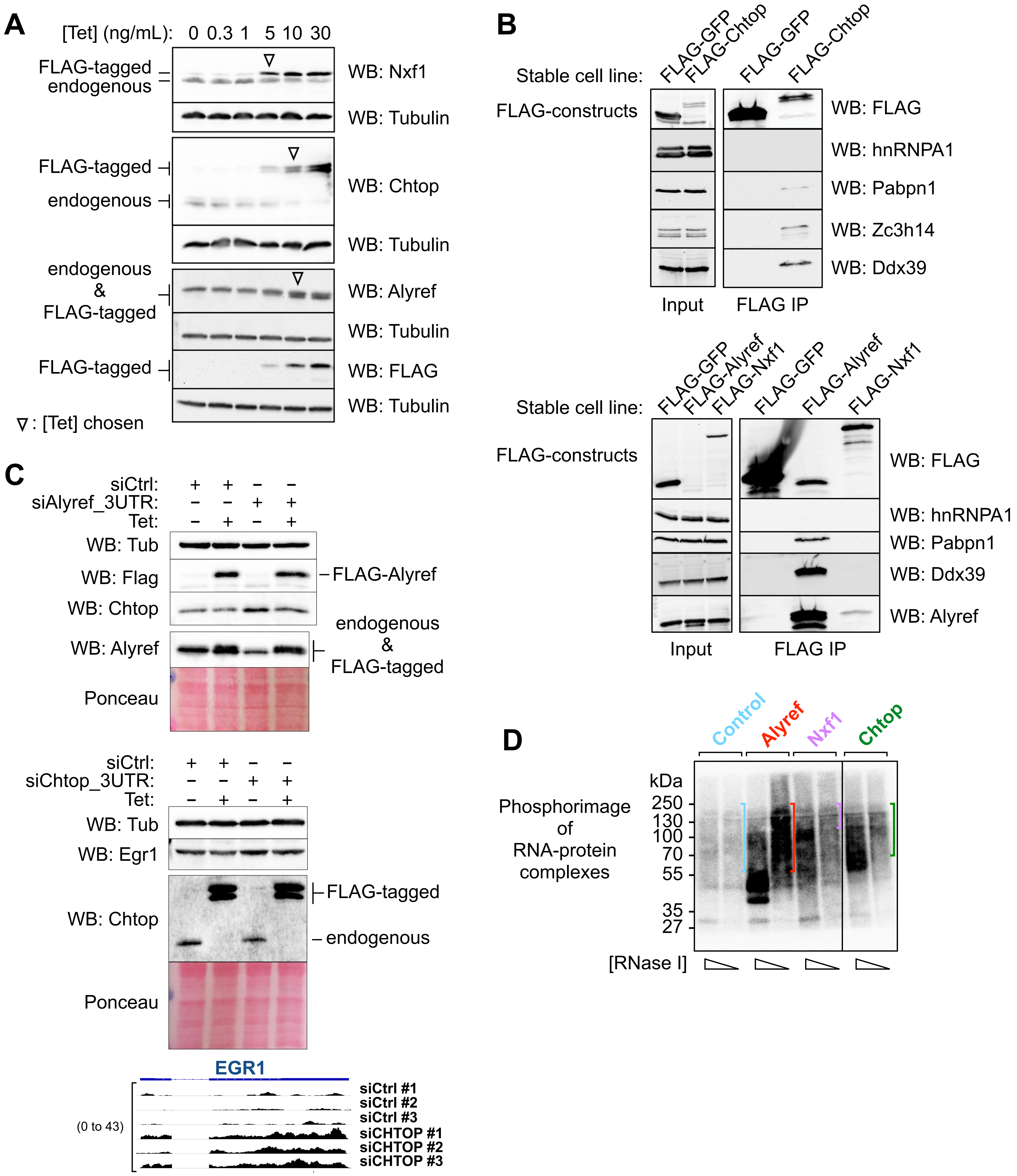

Figure S1. Validations of the FLAG-tagged constructs used for iCLIP. Related to Figure 1. A. Determination of tetracycline concentrations used for iCLIP from cell lines expressing tetracycline-inducible FLAG-tagged Alyref, Chtop, and Nxf1. Open arrowheads indicate the concentration of tetracycline used. **B.** Known interactors co-immunoprecipitate with FLAG-tagged constructs Alyref, Chtop, and Nxf1. hnRNPA1 serves as a negative control (Chang et al., EMBO 2012). **C.** Tetracyline-induced expression of Flag-tagged proteins functionally complements the loss of endogenous proteins by correcting Chtop up-regulation triggered by Alyref knockdown previously described in (Chang et al., EMBO 2012) and Egr1 up-regulation triggered by Chtop knockdown. **D.** Examples of RNA-protein crosslinks obtained during the iCLIP procedure, with coloured brackets indicating range of sizes selected.

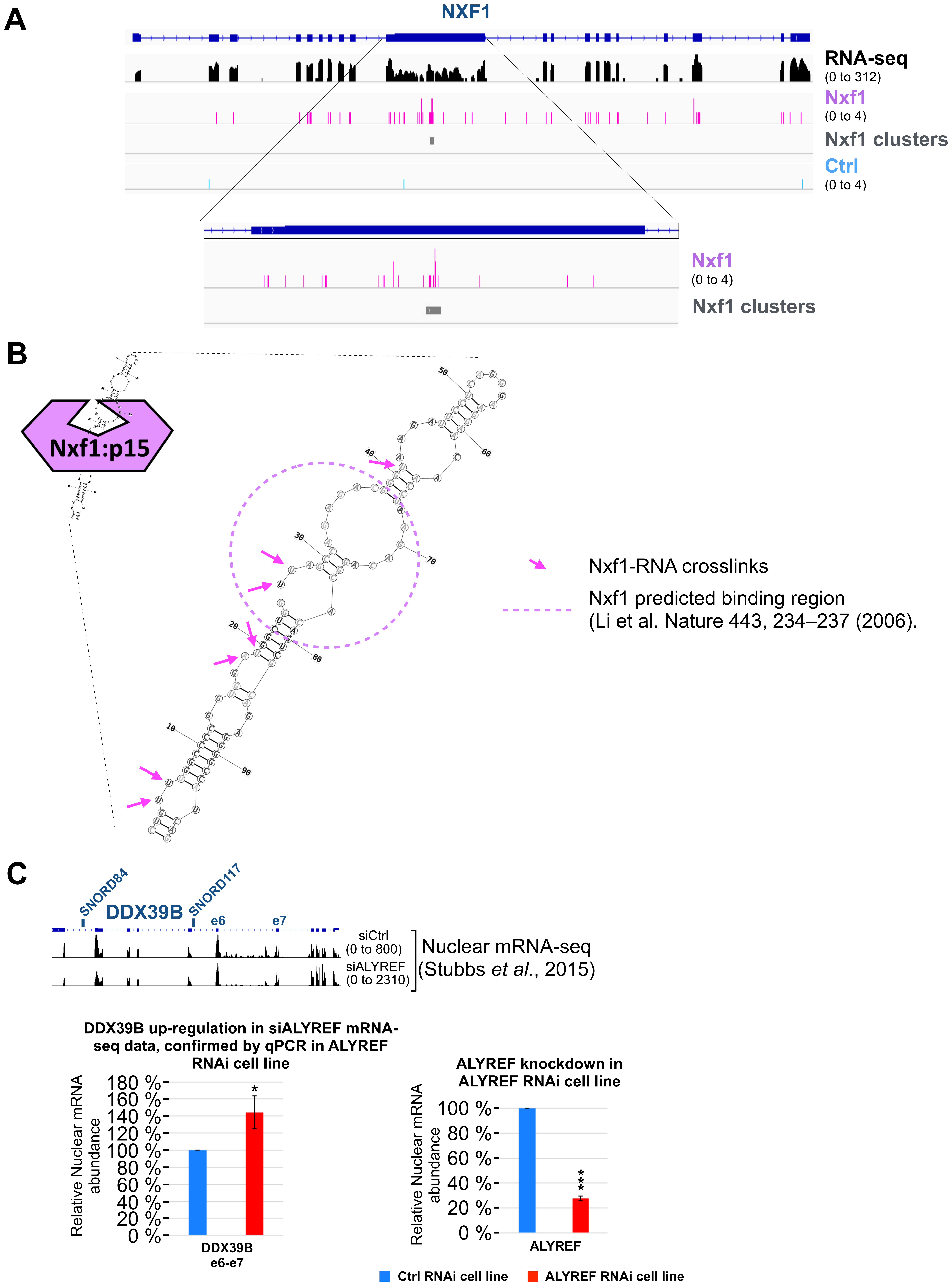

Figure S2. Nxf1 binding to an RNA secondary structure located within intron 10 of its own pre-mRNA, related to Figure 3. Characterisation of Nxf1 binding to intron 10 of its own transcript. **A.** Nxf1 iCLIP confirms the existence of Nxf1 binding cluster within intron 10 of the NXF1 transcript. **B.** Nxf1 crosslinks positions within the CTE-like RNA structure of its intron 10, with indicated predicted Nxf1 binding region on that transcript. **C.** ALYREF knockdown using a different RNAi system and target sequence (Viphakone et al., 2012) reproduces the splicing effect seen in the mRNA-seq data (Stubbs et al., 2015) that we used in our study.

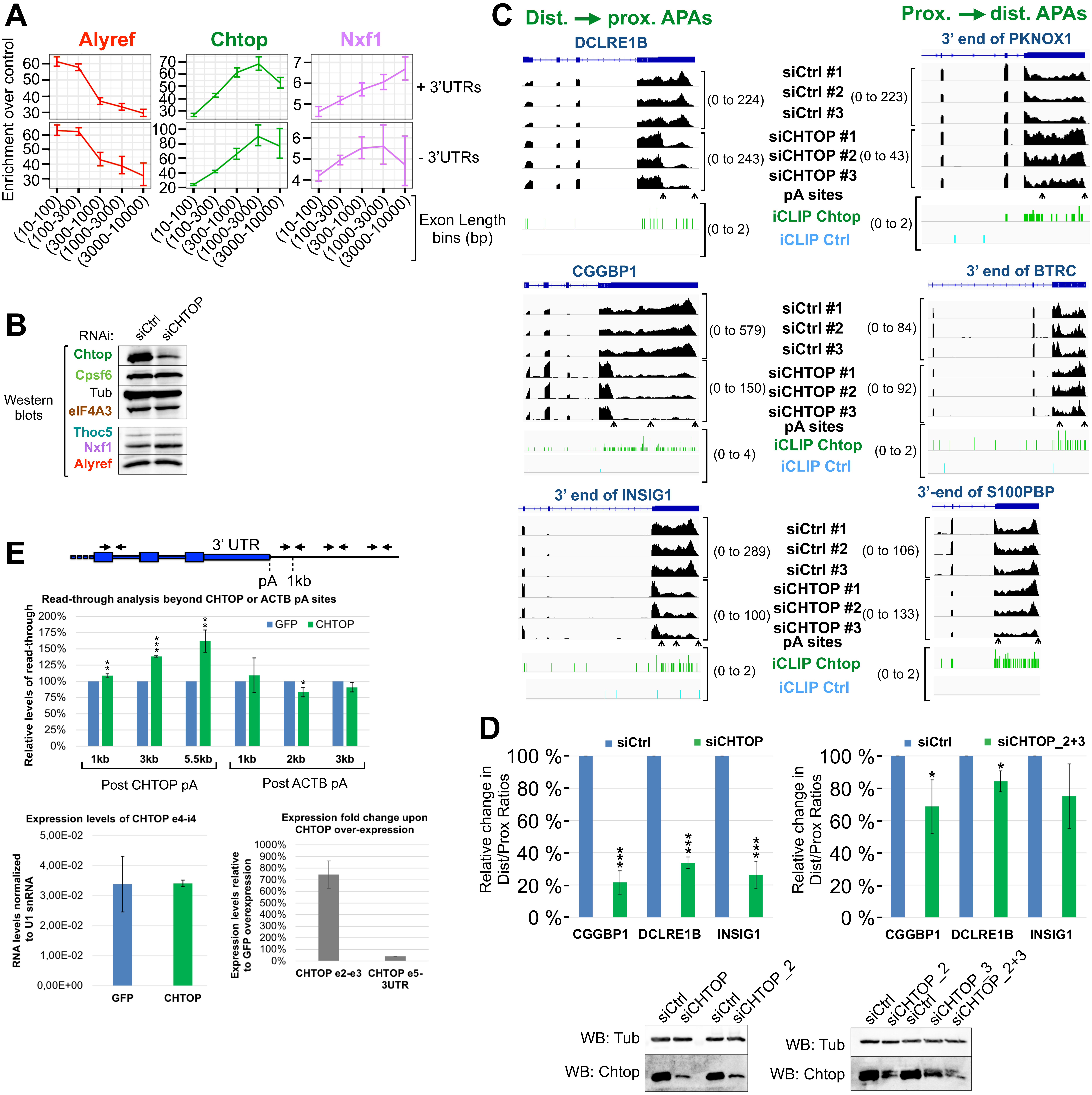

Figure S3. APA and transcription termination regulation by CHTOP. Related to Figure 4. A. Binding enrichment of export factors relative to increasing exon length with 3' UTRs included or not in the analysis. **B.** Western blot analysis of samples used for RNA-seq. 293T cells were subjected to siCHTOP for 72h. **C.** All replicates from Figure 4G and additional examples of Proximal-Distal and Distal-Proximal APA changes. "#number" = replicates. pA sites positions used were from HEK293 A-seq (Martin et al., 2012). **D.** Some APA changes caused by CHTOP knockdown and presented in Figure S3C are validated by qPCR using the siCHTOP siRNA or a less efficient combination of two other siRNAs (siCHTOP_2 and siCHTOP_3). Knockdown efficiencies were assessed by WB analysis. Means of three biological replicates +/- SD. *P< 0.05, **P< 0.01, ***P < 0.001 (t-test). **E.** Read-through transcription beyond CHTOP and ACTB genes was assessed using GFP- or CHTOP-over-expressing stable cell lines. Read-through was normalised to pre-mRNA levels (region e4-i4 in case of CHTOP) and expressed as fold change over signal from GFP-overexpressing cells. Also shown is the down-regulation of endogenous CHTOP mRNA (primers pair "e5-3UTR") upon CHTOP over-expression (primers pair "e2-e3"). Means of three independent experiments +/- SD. *P< 0.05, **P< 0.01, ***P < 0.001 (t-test).

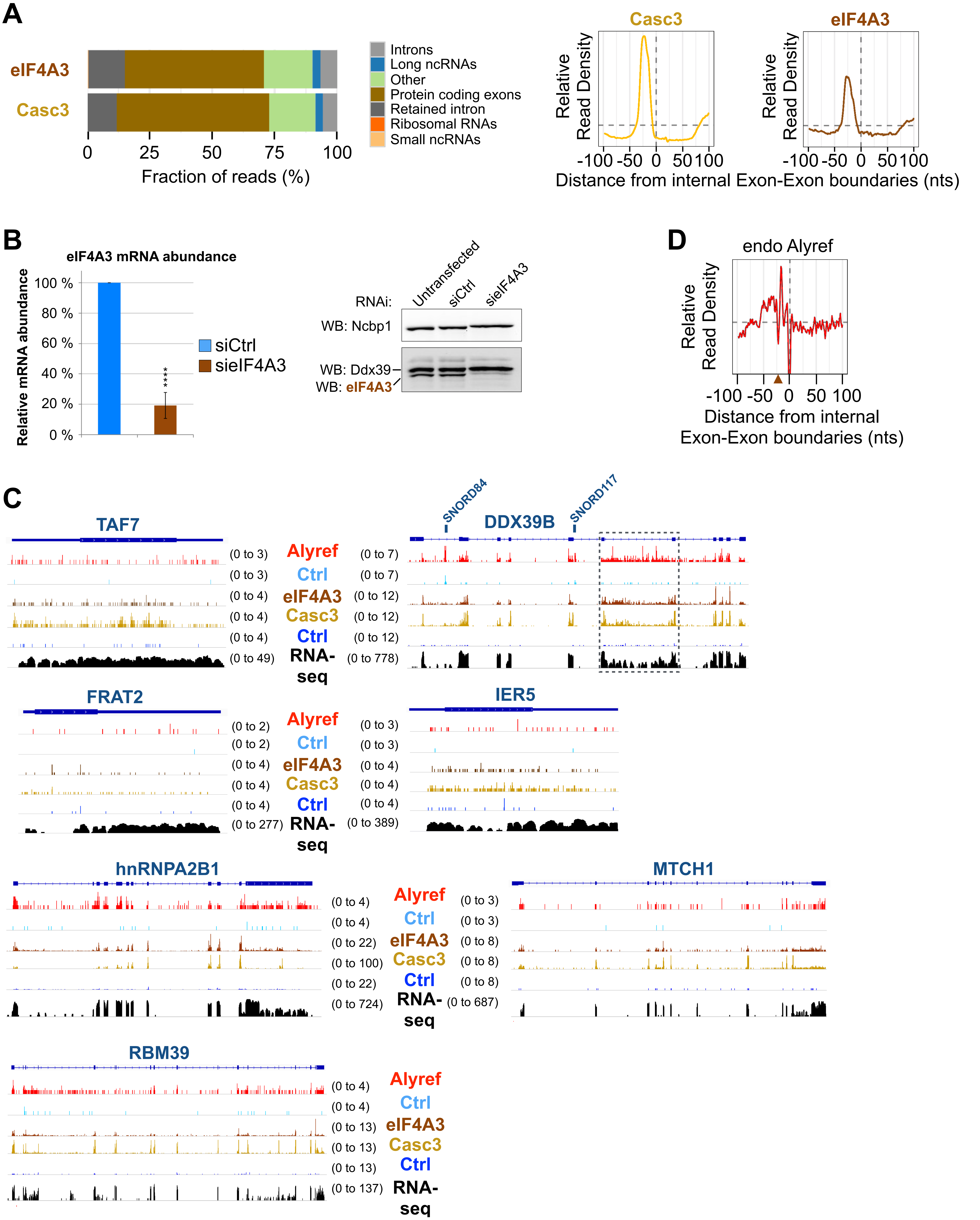

Figure S4. The EJC binds to a variety of RNA species and tethers Alyref. Related to Figure 5. A. Analysis of eIF4A3 and Casc3 iCLIP data from (Hauer et al. 2016). Distribution of reads and metagene analysis at internal exon-exon junctions. **B.** Knockdown efficiency of eIF4A3 analysed by RT-qPCR and Western blot. **C.** Additional iCLIP tracks of the transcripts studied in Figure 5. **D.** Analysis of endogenous Alyref iCLIP data from (Shi et al., 2017). Metagene analysis at internal exon-exon junctions. The EJC's position is inferred from Figure S4A and marked by a brown arrowhead.

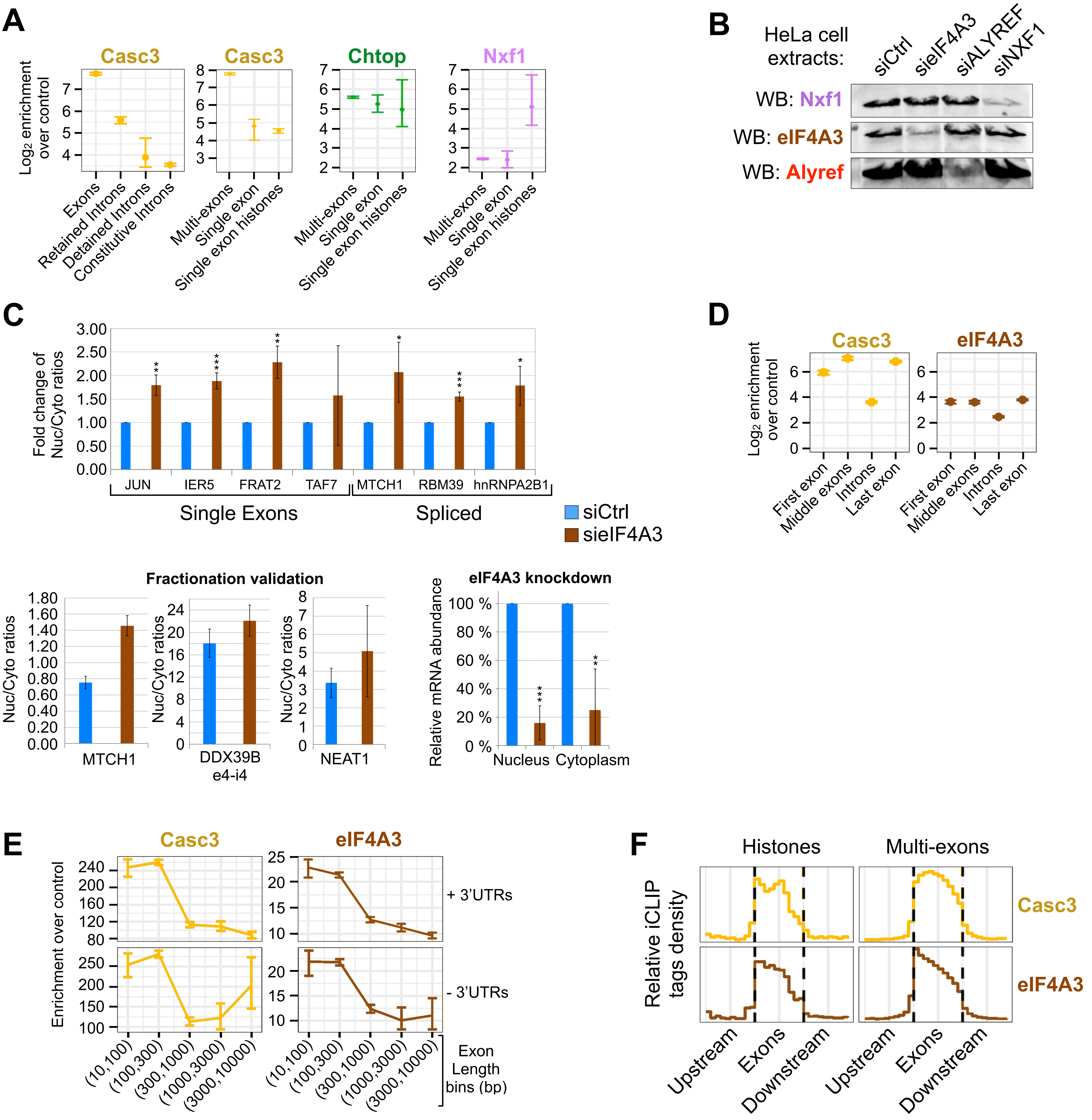

Figure S5. Impact of the EJC on RNA export. Related to Figure 5. A. Log2 enrichment of the indicated factors over their respective iCLIP negative control signals for the indicated genic regions and transcripts. **B.** Knockdown efficiency of RNA export factors in HeLa cells performed alongside the FISH experiment in Figure 5E. **C.** Fold change of Nuclear/Cytoplasmic ratios upon eIF4A3 RNAi in 293T cells analysed by RT-qPCR. Validation of the fractionation was performed by analysing the Nuclear/Cytoplasmic ratios of an mRNA (MTCH1, 0.6 < ratio < 1.5), a pre-mRNA (DDX39B e4-i4, ratio >> 1), and a nuclear retained lncRNA (NEAT1, ratio >1). Means of three independent experiments +/- SD. *P< 0.05, **P< 0.01, ***P < 0.001 (t-test). **D.** Log2 enrichments of the indicated factors over their respective iCLIP negative control signals for the indicated genic regions. **E.** Binding enrichment of EJC core factors over Ctrl iCLIP as a function of increasing exon length with 3' UTRs included or not in the analysis. **F.** Deposition patterns of EJC core factors over single exon histones transcripts as in Figure 4C.

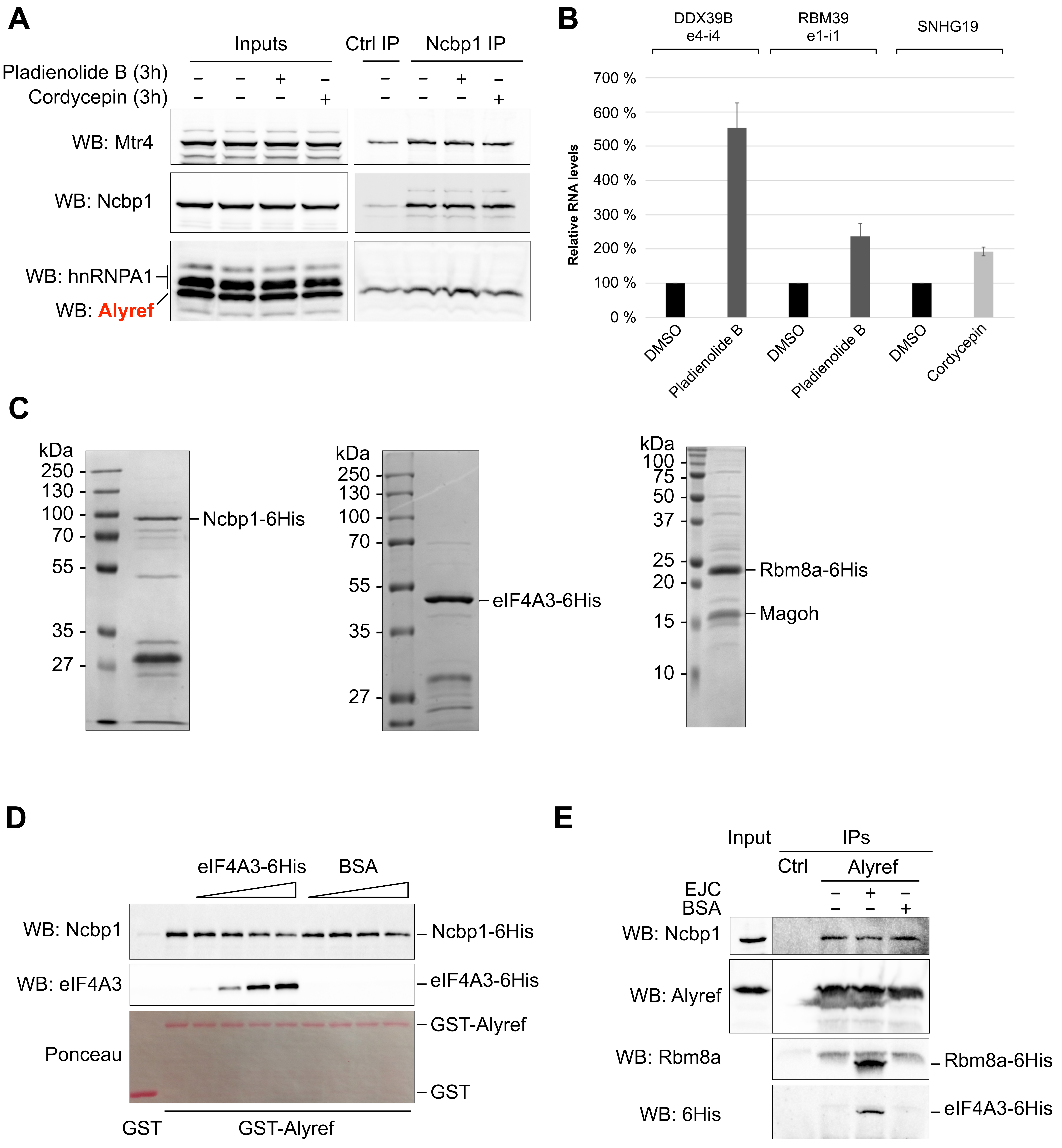

Figure S6. *In vitro* analysis of CBC, EJC, and Alyref interactions. Related to Figure 6. A. Western blot analysis of the co-immunoprecipitation of Alyref with the CBC subunit Ncbp1 from cells treated with the splicing inhibitor pladienolide B or the polyadenylation inhibitor cordycepin. hnRNPA1 is a negative control for interactions with TREX components and shows that RNase A treatment prevented co-IP of general mRNP binding proteins. **B.** The levels of the indicated transcripts were assessed by RT-qPCR in total RNA extracted from 10 % of the cells harvested for IP in Figure S6A. Values are mean +/- SD from two technical replicates. **C.** Recombinant proteins used in Figures S6D and S6F. **D.** GST or GST-Alyref pulldowns using recombinant Ncbp1-6His and increasing concentrations of recombinant eIF4A3-6His or BSA as competitors. **E.** Effect of recombinant EJC or BSA on Ncbp1 co-IP with Alyref.

Oligos pairs name	Forward primer sequence (5' - 3')	Reverse primer sequence (5' - 3')
JUN	GAACTGCACAGCCAGAACAC	TGGGTTGAAGTTGCTGAGG
IER5	TGGCTAACCTCATCAGCATC	GGGTTCATGTCTCTCAGCAC
TAF7	GCCTCTQCTGTGQGQQGGGC	CACGGTCCACTCTGACGATT
FRAT2	AGAAATGTATGCGCCAGGGT	TCGATGCAAGTAGCTGCCAT
DDX39B	CATGCGTCGGGATGTCCAGGAAA	GTCAAAGAGCTTCCGGTTCTTCTCG
DDX39B e6-i6	CATGCGTCGGGATGTCCAGGAAA	CACTTGAATGACAAGGGAGTCTGAGG
DDX39B e4-i4	TGTGTCACACTCGGGAGTTG	ACACCCTCACTCTCAGGTCT
RBM39 e1-e2	CAGCAGCAGCAATCTCTTCC	GAGCCTCAAGCATTGCTTCA
RBM39 e1-i1	CAGCAGCAGCAATCTCTTCC	AATGGAGCCAAACCTCGAGA
ENO1 e3-i3	CTAGAGCTCCGGGACAATGA	ATCATGGGTCACAGCAGGTT
SRSF7 e3-i3	AGATGCTATGAGTGTGGCGA	AAACACTAGTTTCTGCCTTGCT
hnRNPA2B1 e1-e2	GAAATCGGGCTGAAGCGAC	CACACAGTCTGTAAGCTTTCCC
hnRNPA2B1 e1-i1	GAAATCGGGCTGAAGCGAC	ATTTCCTGCCTCTCTCCCAC
hnRNPA2B1	TGATGACCATGATCCTGTGGA	CCTAGAACTCTGAACTTCCTGC
MTCH1	GAGGCCAAGTACAGTGGTGT	GTCATCCACCAGGTAGGCAT
eIF4A3	TTGGATGTCCCTCAGGTGTC	TCTCTGAGGATGCGGATGTC
TARDBP e1-i1	CTGCTTCGGTGTCCCTGT	CAATGCAGAAGCCGAGGC
TARDBP e1-e2	CTGCTTCGGTGTCCCTGT	CAGCCGGACACCTCTCATAC
CHTOP e2-e3	CACCACCAAGATGTCTCTAAATGAGCG	CTGCTTAAGTTTTAATGCTGCCTGGAC
CHTOP e5-3UTR	GAATCGGTGGTAGAGGTCGG	GTCTCTCATGGGAGGATGGG
CHTOP e4-i4	ACGAGGCCTACCCATAATCC	CCATAAGCAGCCCAAGAGAAAAGG
CHTOP 1kb post pA	TGCTCACTACAGTTCCCAGG	TTGTGTTGGTATGGCAAGCC
CHTOP 3kb post pA	AGACCTGACTACCTGCCCTA	CCTCTGTGAGCTCCAAAGGA
CHTOP 5.5kb post pA	TGATGGATGCCAGGCAGTAA	TGCATCTCCATTCAGCAGGA
ACTB unspliced	TCAAGGTGGGTGTCTTTCCT	CCTGCTTGCTGATCCACATC
ACTB 1kb ppA	TGCCTTCCCTCTGCTAGAAG	TGTGCACAGTTGAGAGTCCA
ACTB 2kb ppA	CCAACCAGATGTGTTCCGTG	CAAGACCACCACCACAATCG
ACTB 3kb ppA	AGAGGAAGAGGGCCAGAAAC	TGCAGTGACACAATCTTGGC
NEAT1	GCTTGGAACCTTGCTTCAAG	GGTGGGTAGGTGAGAGGTCA
SNHG19	CGTCCAGGCCTGGCCTAC	GCTCGCGACGAAACCTGC

Table S1. DNA oligonucleotides used in this study. Related to STAR Methods.
