# Supplementary material for: Co-transcriptional Loading of RNA Export Factors Shapes the Human Transcriptome
Source: Mol Cell. 2019 Jul 25;75(2):310–323.e8. doi: 10.1016/j.molcel.2019.04.034 (PMC6675937; doi:10.1016/j.molcel.2019.04.034)
Supplement: Document S2. Article plus Supplemental Information [file mmc2.pdf]

Molecular Cell

Article

Co-transcriptional Loading of RNA Export Factors Shapes the Human Transcriptome

Graphical Abstract

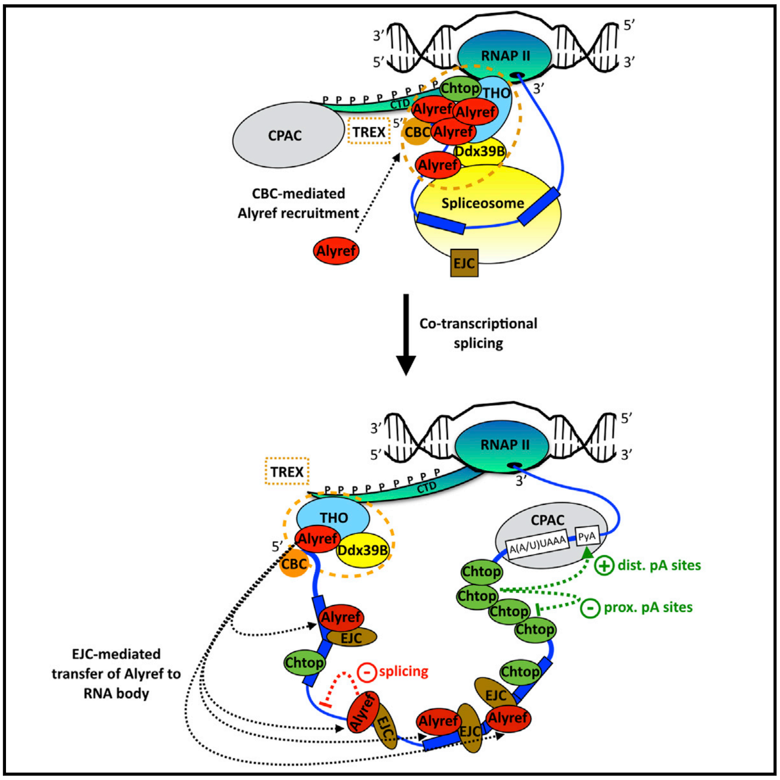

Authors

Nicolas Viphakone, Ian Sudbery, Llywelyn Griffith, Catherine G. Heath, David Sims, Stuart A. Wilson

Correspondence

viphakoneniko@hotmail.com (N.V.),
stuart.wilson@sheffield.ac.uk (S.A.W.)

In Brief

TREX plays a pivotal role in RNA export, though the mechanisms governing its deposition on RNA are obscure. Viphakone et al. show that the cap binding and exon junction complexes drive the ordered deposition of TREX throughout the body of RNA. In doing so, TREX regulates processing of the RNA.

Highlights

- 5′ cap binding complex CBC acts as a transient landing pad for Alyref
- Alyref is deposited upstream of the exon-exon junction next to the EJC
- Alyref can be deposited on introns and regulate splicing
- Chtop is mainly deposited on 3′ UTRs and influences poly(A) site choices

Viphakone et al., 2019, Molecular Cell 75, 310–323
July 25, 2019 © 2019 The Author(s). Published by Elsevier Inc.
https://doi.org/10.1016/j.molcel.2019.04.034

Molecular Cell
Article

Co-transcriptional Loading of RNA Export Factors Shapes the Human Transcriptome

Nicolas Viphakone,[1,3,*] Ian Sudbery,[1,3] Llywelyn Griffith,[1] Catherine G. Heath,[1] David Sims,[2] and Stuart A. Wilson[1,4,*]
[1]Sheffield Institute For Nucleic Acids (SInFoNiA) and Department of Molecular Biology and Biotechnology, The University of Sheffield, Firth Court, Western Bank, Sheffield S10 2TN, UK
[2]MRC Computational Genomics Analysis and Training Programme (CGAT), MRC Centre for Computational Biology, MRC Weatherall Institute of Molecular Medicine, John Radcliffe Hospital, Headington, Oxford, OX3 9DS UK
[3]These authors contributed equally
[4]Lead Contact
*Correspondence: viphakoneniko@hotmail.com (N.V.), stuart.wilson@sheffield.ac.uk (S.A.W.)
https://doi.org/10.1016/j.molcel.2019.04.034

SUMMARY

During gene expression, RNA export factors are mainly known for driving nucleo-cytoplasmic transport. While early studies suggested that the exon junction complex (EJC) provides a binding platform for them, subsequent work proposed that they are only recruited by the cap binding complex to the 5′ end of RNAs, as part of TREX. Using iCLIP, we show that the export receptor Nxf1 and two TREX subunits, Alyref and Chtop, are recruited to the whole mRNA co-transcriptionally via splicing but before 3′ end processing. Consequently, Alyref alters splicing decisions and Chtop regulates alternative polyadenylation. Alyref is recruited to the 5′ end of RNAs by CBC, and our data reveal subsequent binding to RNAs near EJCs. We demonstrate that eIF4A3 stimulates Alyref deposition not only on spliced RNAs close to EJC sites but also on single-exon transcripts. Our study reveals mechanistic insights into the co-transcriptional recruitment of mRNA export factors and how this shapes the human transcriptome.

INTRODUCTION

RNA polymerase II (Pol II) transcribes most human genes as RNA precursors that mature via 5′ capping, splicing, and 3′ end processing (i.e., cleavage and polyadenylation [CPA]), to reach their functional state. This maturation also deposits RNA binding proteins (RBPs) as completion marks for downstream events like RNA nuclear export, localization, translation, and stability (Singh et al., 2015).

Splicing is mostly co-transcriptional in humans (Tilgner et al., 2012), with ~65% of introns removed from nascent RNAs within 5 min (Windhager et al., 2012). Yet up to 80% of pre-mRNAs can be affected by at least one inefficient splicing event (Middleton et al., 2017), causing the retention of 5%–15% of expressed introns within mRNAs (Boutz et al., 2015). Inefficient splicing events are not random, because 32% are conserved from mice to humans. The affected introns are of two types: nuclear-detained introns (DIs), awaiting splicing completion or degradation, and retained introns (RIs) subjected to nonsense-mediated decay (NMD). While intron features influence retention events, the mechanisms involved remain unclear (Boutz et al., 2015).

Following splicing, the exon junction complex (EJC) is deposited 24 nucleotides (nt) upstream of exon-exon junctions (Hauer et al., 2016; Le Hir et al., 2000) as a core of four proteins: the RNA helicase eIF4A3, which anchors the complex on RNA; the heterodimer Rbm8A-Magoh, which locks eIF4A3 in its RNA-bound conformation; and Casc3, which binds RNA and stabilizes the complex (Andersen et al., 2006). The EJC associates dynamically with peripheral proteins mediating its functions in splicing regulation, mRNA translation and stability (Boehm and Gehring, 2016).

As Pol II transcribes the poly(A) site (pA site), the cleavage and polyadenylation complex (CPAC) assembles on pre-RNA to mature its 3′ end. CPA is important for transcription termination, mRNA export, stability, and translation (Eckmann et al., 2011; Fong et al., 2015) and is partly regulated through modulation of cleavage site usage (also termed alternative polyadenylation [APA]). APA affects 70% of human mRNAs (Derti et al., 2012) and regulates cellular proliferation, tumorigenicity, and synaptic plasticity (Tian and Manley, 2017). Various human APA *trans*-regulators have been reported, some directly involved in CPA (Cpsf5, Cpsf6, and Cstf2) (Martin et al., 2012; Masamha et al., 2014; Yao et al., 2012; Zhu et al., 2018), others linked to splicing (hnRNPC, Tardbp, U2af2, and U1 snRNP) (Gruber et al., 2016; Kaida et al., 2010; Millevoi et al., 2006; Rot et al., 2017), and Thoc5 that acts in mRNA export (Katahira et al., 2013).

A major pathway for nuclear mRNA export uses the TREX complex (Strässer et al., 2002), containing a THO subcomplex, the RNA helicase Ddx39b, RNA export adaptors, and co-adaptors that Ddx39b loads onto mRNAs (Heath et al., 2016). A key adaptor is Alyref (Stutz et al., 2000), though some shuttling SR proteins also work as adaptors (Huang et al., 2003). Several co-adaptors have been identified: Chtop, Thoc5, Cpsf6, and Rbm15 (Chang et al., 2013; Katahira et al., 2009; Ruepp et al., 2009; Zolotukhin et al., 2009). These proteins together recruit the export receptor Nxf1 and stimulate its RNA binding activity,

 © 2019 The Author(s). Published by Elsevier Inc.
This is an open access article under the CC BY license (http://creativecommons.org/licenses/by/4.0/).

which promotes mRNA export (Chang et al., 2013; Hautbergue et al., 2008; Viphakone et al., 2015, 2012). The topology of TREX components on RNA and the molecular mechanisms involved in their deposition are still unclear. It has been suggested that the EJC may serve as a binding platform for RNA export factors (Le Hir et al., 2001; Singh et al., 2012). Yet these interactions were not seen in other studies, which instead revealed a key role for the cap binding complex (CBC, containing Ncbp1 and Ncbp2) in recruiting TREX, consistent with the idea that messenger ribonucleoprotein complexes (mRNPs) are exported 5′ end first (Cheng et al., 2006; Chi et al., 2013). Thus, the relative contribution of the EJC and CBC to TREX deposition on the RNA *in vivo* remains unresolved.

Here, we address these outstanding questions by performing individual nucleotide resolution UV crosslinking and immunoprecipitation (iCLIP) (Broughton and Pasquinelli, 2013; König et al., 2011) on the mRNA export factors Alyref, Chtop, and Nxf1. Our *in vivo* results suggest co-transcriptional recruitment of these proteins all along the RNA during splicing but before CPA, which grants them additional roles in gene expression. Alyref can bind inefficiently spliced introns and regulates their splicing, and Chtop binds last exons and participates in APA regulation. We establish the EJC's involvement in recruiting Alyref to spliced and single-exon mRNAs but also to poorly spliced introns *in vivo*. Our data reconcile earlier disparate results by showing that the CBC acts as a transient landing pad for Alyref, which is then transferred during co-transcriptional splicing to sites adjacent to the EJC throughout the RNA.

RESULTS

mRNA Export Factors Bind Protein Coding Transcripts with Specific Deposition Patterns

To gain insight into the RNA species bound by mRNA export factors and their distribution along RNAs *in vivo*, we used iCLIP. We generated stable cell lines expressing near endogenous levels of FLAG-tagged Alyref, Chtop, and Nxf1, which recapitulated known interactions and functionalities (Figure S1) (Fanis et al., 2012; Hautbergue et al., 2008; Chang et al., 2013), allowing us to use the same antibody and identical conditions to permit direct comparisons between iCLIP datasets. Most binding events for all three proteins occurred within long, spliced RNAs, with approximately equal enrichment for classes such as protein coding, large intergenic non-coding RNA (lincRNA), and pseudogenes. In contrast, we observed lower enrichment on short non-coding RNAs, such as small nuclear RNAs (snRNAs) and rRNAs (Figures 1A and 1B). Overall, 91%, 90%, and 84% of the expressed protein-coding transcriptome had at least one CLIP tag for Alyref, Chtop, and Nxf1 respectively and 82% had CLIP tags for all three proteins. We didn't observe clear RNA binding motifs for any of those proteins, even within specific genic regions such as UTRs. This is consistent with the broad binding potential observed and may be related to the use of unstructured arginines to bind RNAs by all three proteins (Chang et al., 2013; Hautbergue et al., 2008).

Among the long non-coding RNAs (lncRNAs) bound by these RNA export factors was XIST (Figure 1C), which is consistent with XIST's interactome (Chu et al., 2015). Because most lncRNAs are chromatin enriched and not exported (Schlackow et al., 2017; Werner and Ruthenburg, 2015), we used previous HEK293T RNA sequencing (RNA-seq) data (Sultan et al., 2014) to identify nuclear and cytoplasmic lncRNAs and examined Alyref, Chtop, and Nxf1 binding to these RNA populations. While Alyref and Chtop were enriched at similar levels regardless of the lncRNAs' location, Nxf1 enrichment was reduced on nuclear lncRNAs compared to cytoplasmic ones (Figure 1D). This may contribute to the nuclear retention of lncRNAs, as previously suggested for XIST (Cohen and Panning, 2007).

Although the three proteins bound along the whole transcript, each had a specific profile. Alyref's distribution displayed a 5′ bias due to strong binding to first exons, consistent with its connection with the CBC (Cheng et al., 2006), but it also bound within the body of the RNA (Figures 1E–1G). Chtop was also found on internal sites, but its binding unexpectedly displayed a 3′ bias (Figures 1E and 1F), which matched a strong enrichment on 3′ UTRs and last exons (Figures 1F and 1G). The RNA export receptor Nxf1 showed a uniform distribution pattern across exons but was enriched on last exons (Figures 1F and 1G), a feature conserved in mouse (Müller-McNicoll et al., 2016). These specific profiles were absent from introns (Figure 1F). Alyref and Nxf1 iCLIP distribution profiles were almost identical to those of their respective yeast orthologs Yra1p and Mex67p obtained by photoactivatable ribonucleoside-enhanced crosslinking and immunoprecipitation (Baejen et al., 2014).

Altogether, these results showed the range of RNAs bound by these TREX components and revealed some intriguing deposition patterns.

RNA Export Factors Are Loaded Co-transcriptionally on Spliced Transcripts before 3′ End Processing

The binding distributions observed within the body of RNAs led us to study links between RNA processing status and recruitment of export factors. We computed a splicing index (SI, the ratio of reads crossing internal exon-exon junctions to those mapping to exon-intron junctions) and a processing index (PI) (as described in Baejen et al., 2014). Alyref, Chtop, and Nxf1 are mostly recruited to spliced but uncleaved RNAs *in vivo* (SI > 1 and PI > 2) (Figures 2A and 2B). While these proteins are known to be nuclear, we found them enriched in the chromatin, together with the TREX subunit Ddx39 (Figure 2C). Moreover, Alyref co-immunoprecipitated (coIP) all forms of Pol II tested, while Chtop preferentially coIP Ser2-phosphorylated Pol II. Nxf1 coIP Pol II, but only in an RNA-dependent manner (Figure 2D). Using chromatin immunoprecipitation sequencing (ChIP-seq), we found Alyref, Chtop, and Nxf1 enriched over gene bodies with patterns similar to the iCLIP profiles (Figures 1F and 2E).

Altogether, these results suggest that RNA export factors are recruited during transcription to the body of the RNA by the splicing process (mostly co-transcriptional in human cells) (Tilgner et al., 2012) but before completion of 3′ end processing.

Alyref Binding to Poorly Spliced Introns Regulates Their Splicing *In Vivo*

Consistent with co-transcriptional recruitment, we also detected binding of RNA export factors to introns (Figure 1A). A known

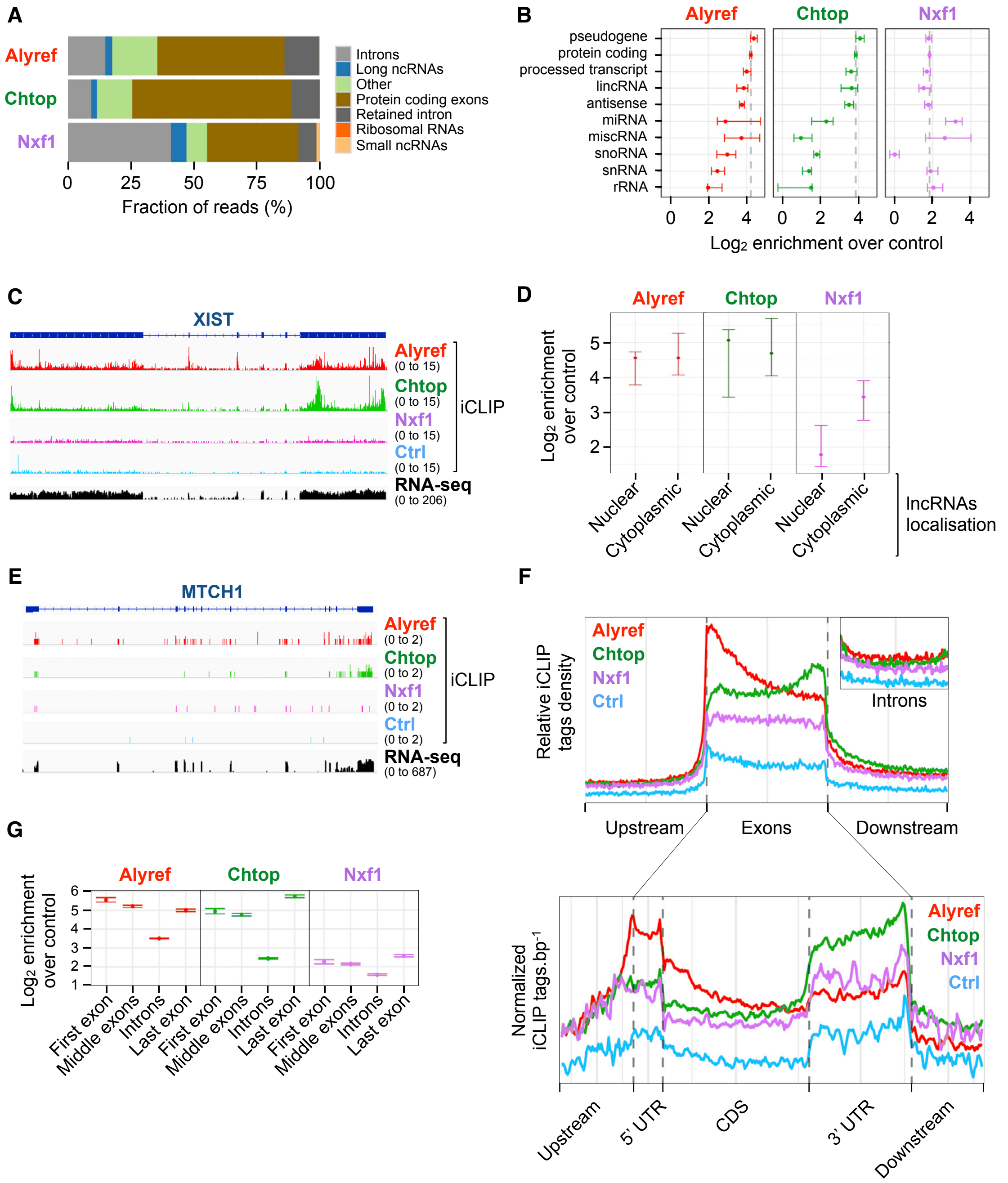

Figure 1. mRNA Export Factors Bind Protein Coding Transcripts with Specific Deposition Patterns

(A) Mapping context of binding clusters found in at least two replicates, but not in control (Ctrl) iCLIP. The "other" category contains miscellaneous RNA (miscRNA), NMD substrates, and processed transcripts.

(B) Binding enrichment of RNA export factors over Ctrl iCLIP for indicated gene biotypes.

(C) RNA export factors binding profiles over XIST lncRNA.

(D) Binding enrichments for nuclear and cytoplasmic lncRNAs.

(E) Example of an mRNA bound by RNA export factors.

(F) Deposition patterns of RNA export factors over indicated genic regions. "Exons" and "CDS" refer to a concatenation of the exonic sequence. Lower-panel data are normalized to RNA expression levels from nuclear RNA-seq.

(G) Binding enrichment over Ctrl iCLIP for first-middle-last exons and introns.

All enrichments are expressed as log_2 crosslinks normalized to the control signal for that class of sequence. Error bars, 95% bootstrap confidence interval. See also Figure S1.

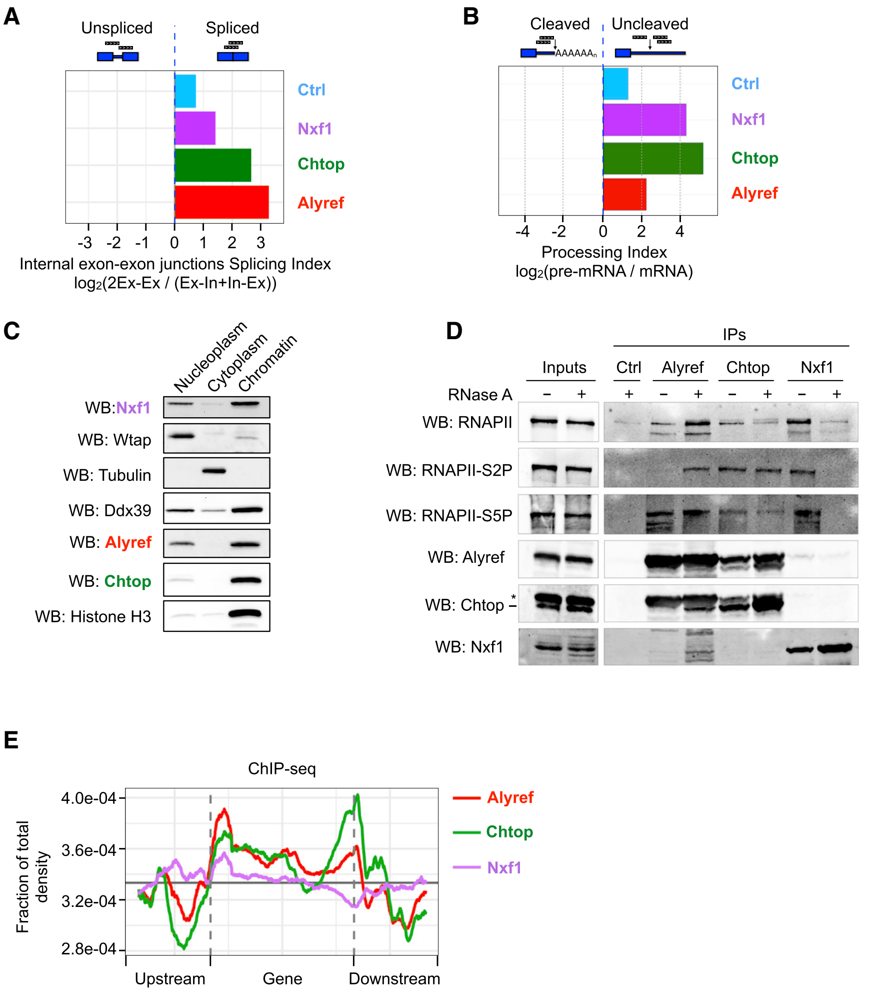

Figure 2. RNA Export Factors Are Loaded Co-transcriptionally on Spliced Transcripts before 3′ End Processing

(A) Splicing indices are calculated as the $\log_2$ ratio of spliced to unspliced reads at exon-exon junctions and indicate a binding preference for spliced versus unspliced RNAs for the designated factors.

(B) Processing indices are calculated as the $\log_2$ ratio of iCLIP read depths for processed and unprocessed RNAs.

(C) Western blot (WB) analysis of 293T cell subcellular fractions.

(D) Pol II forms complexed with RNA export factors *in vivo* analyzed by coIP and WB.

(E) ChIP-seq analysis of RNA export factor distributions along non-overlapping expressed genes.

example of this is Nxf1 binding to an RNA secondary structure located within intron 10 of its own pre-mRNA (Li et al., 2006), which our data recapitulated (Figures S2A and S2B). To look more closely at intronic binding events, we defined RIs, DIs, and introns not falling into these two groups in HEK293T cells (see STAR Methods and Boutz et al., 2015). While the splicing factor Ptbp1 was mainly enriched on constitutive introns, export factors bound all transcript regions, including constitutive introns, but were even more enriched on inefficiently spliced DIs and RIs (but not to the level of exonic sequences), with the adaptor Alyref displaying the strongest enrichment (Figures 3A and 3B).

To determine whether Alyref binding to poorly spliced introns was functionally relevant, we studied the effect of Alyref RNAi on RI and DI splicing using nuclear mRNA sequencing (mRNA-seq) data from HEK293T cells (Stubbs and Conrad, 2015). We used DEXseq to compare changes in the RNA-seq signal over each intron with changes in other areas of their host transcript. We found that relative levels of up to 18% of DIs and 12% of RIs bound by Alyref were reduced upon Alyref RNAi compared to <2% of other transcript regions (Figure 3C), either by an effect on splicing or on the relative stability of spliced versus unspliced isoforms. Among these more efficiently spliced RNAs was DDX39B, and this was replicated with another RNAi targeting sequence (Figure S2C). This indicates that Alyref can also bind poorly spliced introns and influence splicing outcomes.

Chtop Participates in APA Regulation *In Vivo*

The co-transcriptional recruitment of Chtop was also particularly interesting for several reasons. Firstly, its high PI (> 4) (Figure 2B) suggested loading before 3′ end cleavage. Secondly, Chtop coIP Ser2-phosphorylated Pol II, a form enriched at the 3′ end of genes and important for 3′ end processing, and Chtop was recruited at the 3′ end of genes (Figure 2E). Thirdly, Chtop binding events on 3′ UTRs were not affected by gene length and occurred within 1.5 kb of the 3′ end of the gene (Figure 4A), which matches the average size of 3′ UTRs in humans. By analyzing Chtop binding as a function of exon length, we found it preferentially bound long exons, unlike Alyref (Figure 4B). This probably explains the binding of Chtop to 3′ UTRs and last exons, which are on average the longest exons in transcripts. This trend persisted even when 3′ UTRs were excluded from the analysis (Figure S3A). Fourthly, the 3′ bias observed in Chtop binding profile was absent on replication-dependent histone mRNAs (Figure 4C), which are not usually polyadenylated in metazoans (Romeo and Schümperli, 2016). Finally, Chtop binds to the NTF2-like domain of Nxf1 (Chang et al., 2013), and all other confirmed or putative co-adaptors known to bind to this domain have been implicated in APA regulation (Katahira et al., 2013; Ruepp et al., 2009) (Figure 4D).

These results led us to explore whether Chtop might regulate APA *in vivo* using RNA-seq on poly(A)+ RNAs from HEK293T cells depleted for Chtop. Chtop RNAi didn't change the levels of Cpsf6 or Thoc5, both important APA regulators (Figure S3B). We observed 432 APA changes distributed as follows: a global reduction in distal pA site usage (Figure 4E,

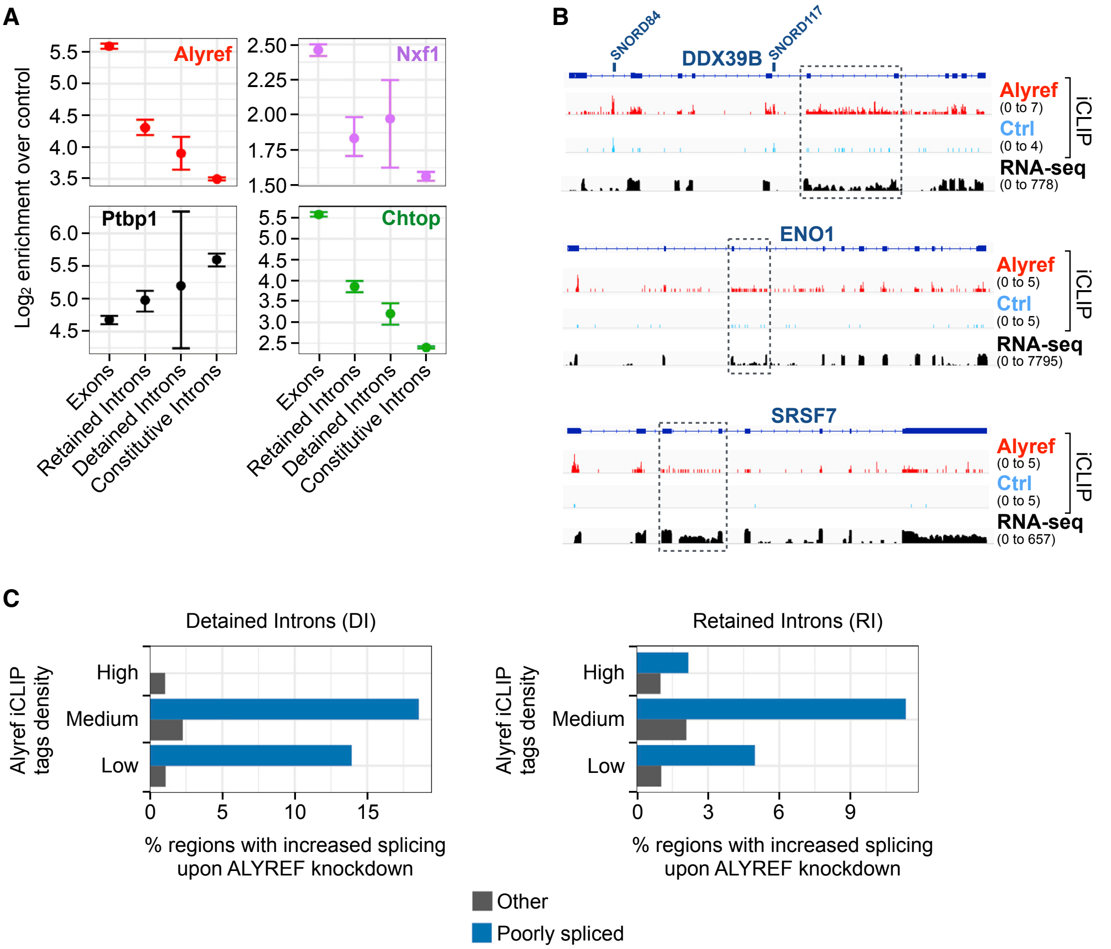

Figure 3. Alyref Binding to Poorly Spliced Introns Regulates Their Splicing *In Vivo*

(A) Binding enrichment of RNA export factors and splicing factor Ptbp1 for indicated RNA regions, calculated and displayed as in Figure 1.

(B) Examples of transcripts showing Alyref CLIP tags within introns that are normally inefficiently spliced (dashed boxes).

(C) Fraction of RIs, DIs, and other transcript regions segregated by Alyref binding status and called as differentially included upon ALYREF RNAi using DEXSeq (false discovery rate [FDR] 10%, fold change > 1.5).

See also Figure S2.

gray curve shifted to the left) with 367 distal-to-proximal and 61 proximal-to-distal significant changes. As an additional approach, we calculated transcript-specific expression levels and measured the per gene expression-weighted average transcript length. We found that 892 genes produced shorter transcripts and 276 genes produced longer transcripts upon Chtop RNAi (Figure 4F). No correlation was seen between APA events and expression levels (R^2 = 0.004), suggesting this effect wasn't due to changes in RNA stability. Examples of APA changes triggered by Chtop RNAi are shown in Figures 4G and S3C, and some were confirmed by qPCR and reproduced with other small interfering RNAs (siRNAs), but to a lower extent due to a lower RNAi efficiency (Figure S3D). As a reference, Cpsf6 RNAi in HEK293T has been reported to trigger 775 significant APA changes (718 distal to proximal and 57 proximal to distal) (Martin et al., 2012, with data reanalyzed by Zhu et al., 2018). Pre-RNA 3′ end processing is intimately linked to transcription termination (Fong et al., 2015), and Chtop overexpression leads to transcriptional read-through downstream of the CHTOP gene (Figure S3E).

Overall, these results suggest that co-transcriptional recruitment of the RNA export factor Chtop to 3′ UTRs and last exons participates in defining the pattern of pA sites used *in vivo*.

eIF4A3 Is Important for Alyref Binding to RNA *In Vivo* and mRNA Export

The presence of RNA export factors on the body of RNAs led us to look at their distribution at internal exon-exon junctions. As a reference, we reanalyzed the EJC iCLIP data from Hauer et al. (2016) and confirmed that the EJC globally covers a region between −50 and −5 nt upstream of the exon-exon junction, peaking at −24 nt (Figure S4A). We observed enrichment of Alyref in a region spanning −75 to −24 nt upstream of the exon-exon junction, with a peak around −37 nt (Figure 5A). In contrast, this specific pattern was not observed for Chtop or Nxf1. We detected similar Alyref enrichment in endogenous iCLIP data from Shi

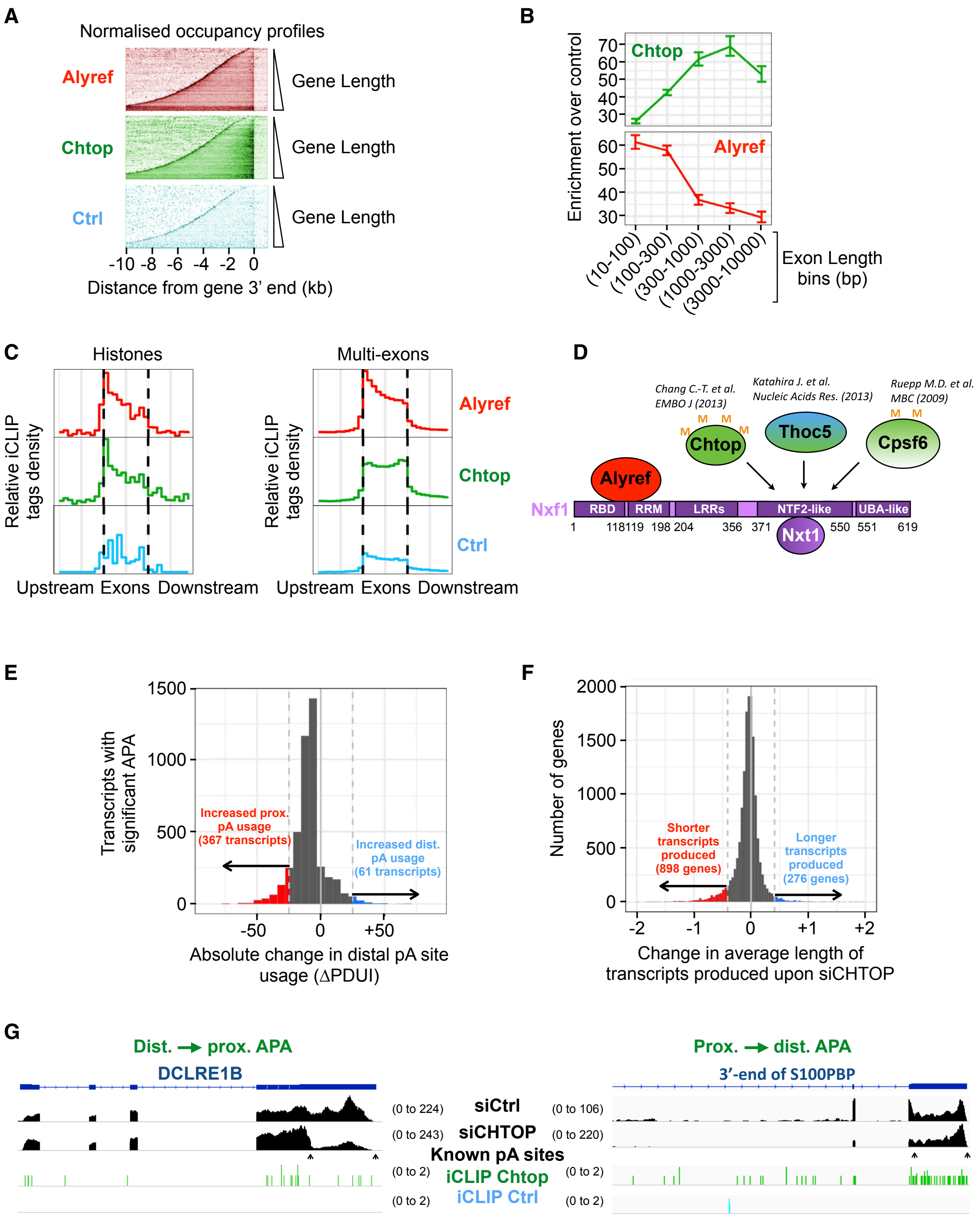

Figure 4. Chtop Participates in Poly(A) Site Choice *In Vivo*

(A) Occupancy profiles for indicated factors and Ctrl iCLIP on transcripts produced from genes sorted by increasing length and aligned at their 3′ ends and normalized to nuclear RNA levels.

(B) Binding enrichment of Alyref and Chtop over Ctrl iCLIP as a function of increasing exon length with 95% bootstrap confidence intervals.

(C) Deposition pattern of Alyref and Chtop over single-exon histones and multi-exons RNAs using similar bins.

(D) Co-adaptors (in shades of green) known to bind to Nxf1's NTF2L domain. Thoc5 and Cpsf6 have been shown to regulate APA in the indicated articles.

(E) Histogram showing the distribution of change in the Percentage of Distal pA site Usage Index (ΔPDUI) for RNAs found to have significant APA upon Chtop RNAi (5% FDR) using the DaPars package. Colored regions show transcripts where |ΔPDUI| > 25%.

(legend continued on next page)

et al. (2017) (Figure S4D), which showed our FLAG-Alyref construct reproduced genuine Alyref behavior transcriptome-wide. Given the EJC's known position (Figures 5A, brown arrowheads, and S4A), this suggests that Alyref binds closely to the EJC within those internal regions of the RNA, as illustrated in Figure 5A.

The juxtaposition of Alyref and the EJC at exon-exon junctions and the earlier description of Alyref as a peripheral EJC component (Le Hir et al., 2000) led us to test the EJC's importance for Alyref deposition at spliced junctions *in vivo*. Thus, we performed RNA immunoprecipitation (RIP) on Alyref using cells depleted of eIF4A3, the EJC's anchor. eIF4A3 RNAi efficiency was assessed by western blot and qRT-PCR in the RIP samples (Figure S4B). eIF4A3 RNAi led to a reduction in the amount of not only spliced RNAs that coIP with Alyref but also unspliced RNAs (Figure 5B), which Alyref can bind (Figure 3A). We initially included single-exon transcripts as negative controls in these RIPs. To our surprise, eIF4A3 RNAi also severely reduced the amount of RNAs that coIP with Alyref (Figure 5B). In agreement with these effects, we found the core EJC components eIF4A3 and Casc3 bound to these inefficiently spliced introns and to single-exon transcripts (Figures 5C and S4C). Globally, these two core EJC subunits were enriched on RIs and DIs over constitutive introns, albeit at a lower level than on exons, especially in the case of Casc3. They were also enriched on histone and non-histone single-exon RNAs, but less than on multi-exons (Figures 5D and S5A). Consequently, we tested whether eIF4A3 plays a role in mRNA export in human cells. eIF4A3 RNAi led to moderate nuclear accumulation of poly(A)+ RNAs as assessed by oligo(dT) fluorescence *in situ* hybridization (FISH), compared to the effects caused by depleting the export adaptor Alyref or the export receptor Nxf1 (Figures 5E and S5B). The strength of this phenotype was similar to that reported for Acinus, another EJC component (Chi et al., 2014). eIF4A3 RNAi also led to an increase in the nucleo-cytoplasmic ratio of both single-exon and spliced mRNAs (Figure S5C), consistent with defective nuclear export.

Altogether, these results highlight the EJC's importance for Alyref deposition onto an unexpected variety of transcripts and for the export of single-exon and spliced RNAs.

CBC Acts as a Transient Landing Pad for Alyref

Because the EJC is mostly present on open reading frames (ORFs) or middle exons (Hauer et al., 2016) (confirmed in Figure S5D), its significant role in recruiting Alyref that we discovered was puzzling given the Alyref 5′ enrichment (Figures 1F and 1G). Because the 5′ mRNA cap is added soon after transcription initiation, Alyref 5′ bias and its known association with the CBC (Cheng et al., 2006) would suggest that Alyref could bind early to the 5′ end, possibly before excision of the first intron of the transcript. However, we found by RIPs that Alyref preferentially bound to 5′ ends that had already undergone splicing (Figure 6A). To analyze this globally, we computed a SI restricted to the first exon-exon junctions and found that Alyref preferentially binds to spliced RNAs, even at the 5′ end (Figure 6B). These results suggested that Alyref could be tethered at the 5′ end of RNAs by the CBC but that it still required splicing for binding, as previously proposed (Cheng et al., 2006).

In an attempt to explain these discrepancies, we hypothesized that after a pre-requisite initial recruitment of Alyref by the EJC, the spatial proximity between EJCs and the CBC, induced by EJC-mediated compaction of the mRNP (Singh et al., 2012), might allow a transfer of Alyref to the CBC. To test this, we performed IP on CBC and looked at the levels of Alyref co-purifying in an eIF4A3 RNAi context (Figure 6C). If this hypothesis was true, then eIF4A3 RNAi should result in a reduced amount of Alyref bound to CBC. Surprisingly, we observed the opposite: Alyref specifically accumulated on the CBC upon eIF4A3 RNAi (Figure 6D). We then performed CBC IPs after transcription inhibition by actinomycin D or 5,6-dichloro-1-β-D-ribofuranosyl-1H-benzimidazole (DRB), which also led to accumulation of Alyref on CBC (Figures 6C and 6E). The same phenomenon was observed upon splicing inhibition with pladienolide B, whereas treatment with cordycepin, an inhibitor of polyadenylation, had no effect (Figures 6C and S6A). To confirm that the two drugs were active in this experiment, we performed qRT-PCR on total RNA extracted from a fraction of the IP inputs. Splicing was inhibited, as shown by an increase in some unspliced RNAs, and cordycepin treatment led to an expected increase of the PAXT complex RNA target SNHG19, as observed previously (Meola et al., 2016) (Figure S6B). These *in vivo* results show that interfering with co-transcriptional recruitment of Alyref can lead to its accumulation on the CBC.

A study found that mutating a WxHD motif in Alyref abrogates the interaction of FLAG-tagged Alyref with both Ncbp1 and eIF4A3, suggestive of a mutually exclusive site (Gromadzka et al., 2016). To directly test this, we pulled down recombinant Ncbp1 using glutathione S-transferase (GST)-Alyref and used purified eIF4A3 or BSA as competitors (Figures S6C and S6D). eIF4A3 could partially compete Ncbp1 binding to GST-Alyref. We also analyzed the ability of a mixture of recombinant core EJC proteins (eIF4A3 and Rbm8A-Magoh) to displace Ncbp1 from Alyref after IP. Again, purified EJC proteins could partially compete with Ncbp1 for binding to Alyref (Figures S6C and S6E).

Overall, in combination with our iCLIP data, these experiments suggest that *in vivo*, Alyref binds to the CBC initially but transiently, before being subsequently deposited near the EJC on the RNA.

DISCUSSION

From our data, we propose a model for the co-transcriptional recruitment of RNA export factors and their influence on gene expression *in vivo* (Figure 7) that unifies two long-standing views: a CBC-only model or an EJC-centric model. As the RNA exits Pol II, the CBC elicits early recruitment of mRNA export factors at the 5′ end. As components of the spliceosome rapidly scan the RNA,

(F) Histogram of the $\log_2$ fold change in average effective gene length upon Chtop RNAi, calculated with the TxImport package as an average of transcript lengths weighted by transcript usage.

(G) Examples of APA changes observed upon Chtop RNAi. RNA-seq tracks are shown in black. pA site positions are from HEK293 A-seq (Martin et al., 2012). See also Figure S3.

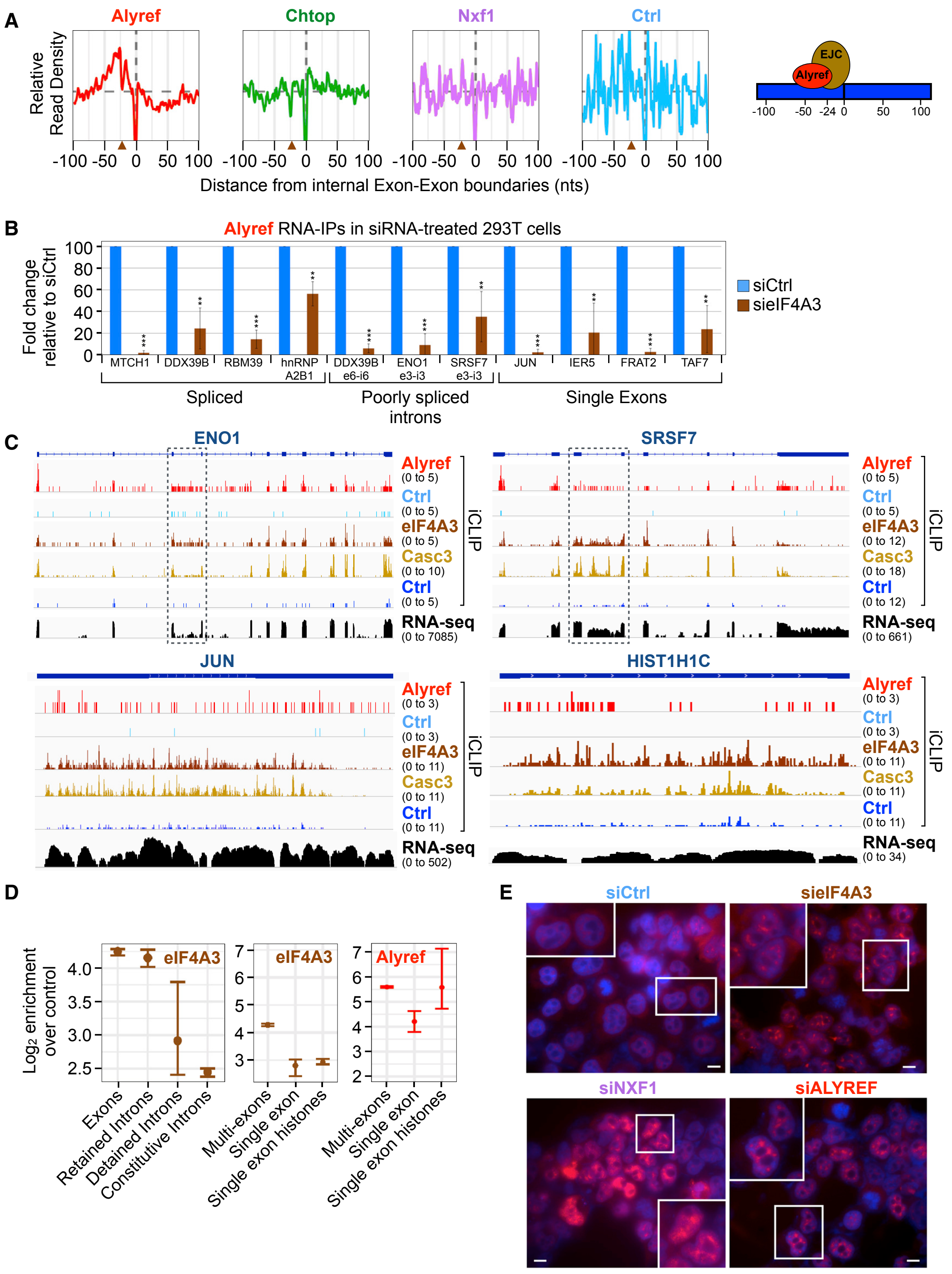

Figure 5. eIF4A3 Is Important for Alyref Binding to Various RNAs and mRNA Export

(A) RNA export factors average binding profiles at internal exon-exon junctions. EJC's position is inferred from Figure S4A and marked by brown arrowheads.

(B) Relative changes in Alyref RIP efficiency upon eIF4A3 RNAi in 293T cells. Mean of three independent experiments ± SD. $^{**}p < 0.01$, $^{***}p < 0.001$ (t test).

(legend continued on next page)

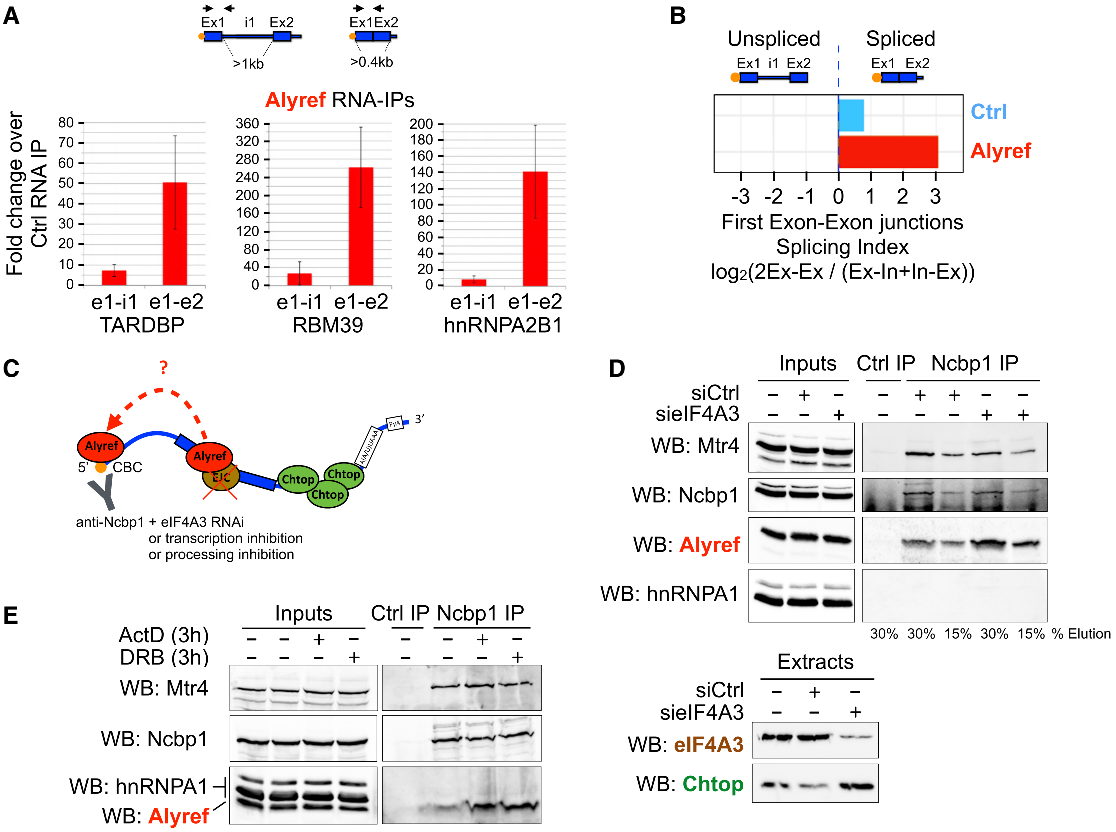

Figure 6. CBC Acts as a Transient Landing Pad for Alyref

(A) Influence of splicing on endogenous Alyref binding to the 5′ end of selected transcripts by RIP. Means of three independent experiments ± SD.

(B) Splicing indices at first exon-exon junctions computed for Alyref and iCLIP Ctrl as in Figure 2.

(C) Experimental strategy used in (D) and (E).

(D) Effect of eIF4A3 RNAi on Alyref coIP with the CBC subunit Ncbp1 analyzed by WB. hnRNPA1 is a negative control. Mtr4 is a known CBC interactor. Efficient eIF4A3 RNAi is shown in the lower panel.

(E) Effect of transcription inhibition using actinomycin D (ActD) or DRB on Alyref coIP with Ncbp1 analyzed by WB.

See also Figure S6.

this may suffice to deposit the EJC and mRNA export factors on single-exon transcripts but at a lower efficiency than on intron-containing RNAs (Figures 5D and S5A). On multi-exon transcripts, splicing deposits the EJC. Alyref then transfers from the CBC to exon-exon junctions along the RNA, upstream of the EJC. Intron retention can also trigger atypical binding of the EJC and Alyref, which in turn regulates this phenomenon. As the last exon is produced by Pol II, Chtop presence increases and it participates in APA regulation. Ultimately, Alyref and Chtop allow Nxf1 to join the RNP and stimulate nuclear export.

Because TREX and the EJC can be loaded on RNAs via splicing but independently of transcription *in vitro* (Cheng et al., 2006), we cannot rule out that some Alyref may be loaded post-transcriptionally. However, this probably accounts for a minority of events, because splicing is mostly co-transcriptional in human cells. Even though our data could reflect an influence of the CBC in the splicing of the first intron (Ohno et al., 1987), our model is supported by the following lines of evidence. Alyref associates with spliced RNAs in a cap- and EJC-dependent manner *in vitro*, but simultaneous binding of Alyref to both CBC and EJC seems unlikely *in vivo* (Gromadzka et al., 2016) (Figures 6E and S6D). Moreover, the CBC isn't present in the EJC's interactome (Singh et al., 2012). Consistent with Alyref being only transiently associated with CBC *in vivo*, Alyref isn't strongly enriched in CBC interactome studies at steady state, unlike Mtr4 (also known as Skivl2) or THO components (Andersen et al., 2013; Gebhardt et al., 2015; Pabis et al., 2013). Furthermore, we found Alyref bound all along the RNA (Figures 1F and 1G), and depletion of the CBC subunit Ncbp1 decreases Alyref binding not only at the 5′ end but also throughout the body

(C) Examples of EJC bound to intronic (dashed boxes) or single-exon RNAs. Also see Figure S4C.

(D) eIF4A3 and Alyref binding enrichments over Ctrl iCLIP on indicated RNA types and regions (as in Figure 1).

(E) Oligo(dT) FISH on HeLa cells RNAi for the indicated factors. Pictures were taken at the same exposure level. White boxes indicate higher magnification. Scale bar, 10 μm. See also Figures S4 and S5.

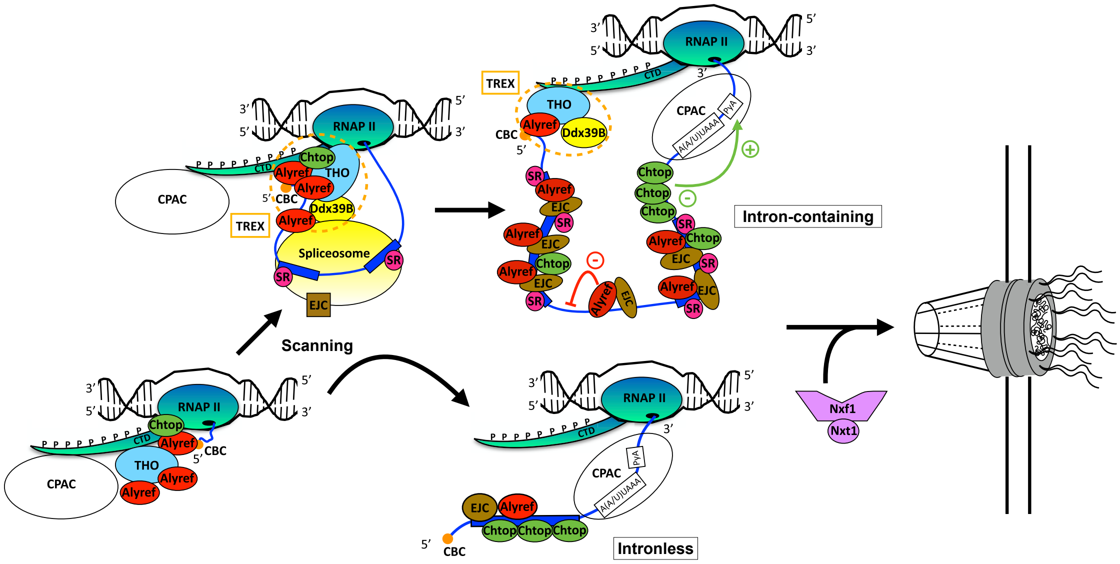

Figure 7. Co-transcriptional Recruitment of mRNA Export Factors and Its Effect on Gene Expression
Model for the co-transcriptional recruitment of RNA export factors and their influence on gene expression *in vivo*.

of the RNA (Shi et al., 2017). Moreover, the CBC appears to be critical for poly(A)+ RNA export in human cells, contrary to the findings of previous studies (Gebhardt et al., 2015). The repeated loading of Alyref from CBC onto internal sites juxtaposed with EJCs would ensure that the CBC remains loaded with Alyref throughout mRNP maturation. This is likely to be important in mRNP quality control as Alyref competes with Mtr4 for CBC interaction, which may determine whether an mRNA is exported or degraded by the exosome (Fan et al., 2017).

The co-transcriptional nature of the recruitment of RNA export factors has several implications. Firstly, it shows that Alyref binding to poorly spliced introns participates in regulating their presence in mature transcripts transcriptome-wide (Figure 3). Although initially considered a biological defect, intron retention is now regarded as part of crucial regulatory programs activated during physiological or pathological gene expression, such as cellular differentiation (Llorian et al., 2016), response to neuronal activity (Mauger et al., 2016), and tumor suppressor inactivation in cancer (Jung et al., 2015). Our findings suggest that canonical mRNA export factors have the potential to play a role in these processes.

Secondly, co-transcriptional recruitment raises questions regarding the various signals required to release the RNP from the chromatin for nuclear export. Nxf1's presence is likely to be one of those signals, given its reduced levels on nuclear-retained lncRNAs (Figure 1D) and the export competence that it grants even to unspliced transcripts (Braun et al., 2001; Li et al., 2006). Yet the binding of RNA export factors to poorly spliced introns (Figure 3) is overall clearly insufficient, because only 10% of RI-containing transcripts induced by splicing inhibition reach the cytoplasm (Yoshimoto et al., 2017). Another important signal is probably the RNA's maturation state, because inefficient processing impairs release from the site of transcription and leads to degradation by the nuclear exosome (Custódio et al., 1999; Schlackow et al., 2017). Alyref loading at the 5′ end helps RNAs evade nuclear degradation by the exosome (Fan et al., 2017). In mammals, there is growing evidence that splicing more than 3′ end formation is responsible for the nuclear retention of transcripts and their release for export. Poly(A)+ RNAs can be found at transcription sites after transcription termination, and splicing completion can delay the release of fully 3′ end-formed RNAs from the chromatin (Brody et al., 2011; Mauger et al., 2016; Pandya-Jones et al., 2013). Moreover, lncRNAs are mainly associated with chromatin (Werner and Ruthenburg, 2015), and splicing more than 3′ end formation seems to define their poor processing state, leading to degradation by the nuclear exosome (Schlackow et al., 2017). In line with this, we show that the splicing process is key to allowing co-transcriptional recruitment of export factors (Figure 2) and that unspliced species recruit export factors less efficiently (Figure 3A, 5D, and 6A). These results are consistent with the roles played by splicing and TREX components in releasing spliced RNAs from nuclear speckles and stimulating mRNA export (Dias et al., 2010).

Thirdly, our data on Chtop participating in APA regulation (Figure 4) support the view that the co-transcriptional loading of RNA export factors can influence 3′ end processing in human cells. Because Cpsf6 is a master regulator of APA in the cell, we looked at the set of transcripts displaying APA changes in both siCPSF6 (Martin et al., 2012) and siCHTOP conditions. It was restricted to only 21 transcripts, revealing the complex interplay taking place on the last exons to establish the pattern of pA sites used in the cell. The broad distribution of Chtop on the last exons might assist with restricting the spatial deposition of the CPAC. Nxf1 was upregulated in response to Chtop RNAi (Figure S3B), and Nxf1 RNAi triggers hyperadenylation of reporter transcripts (Qu et al., 2009). These results reinforce the functional connection we have previously uncovered between Nxf1 and Chtop (Chang

et al., 2013). Consistent with involvement of Chtop in 3′ end-processing regulation, it preferentially coIP Ser2-phosphorylated Pol II (Figure 2D), and its overexpression specifically led to transcriptional readthrough downstream of its own gene (Figure S3E). Perhaps this constitutes another mechanism used by Chtop to auto-regulate its expression (Figures S1A and S3E) (Izumikawa et al., 2016).

Although the EJC wasn't originally implicated in RNA export, those data were obtained either *in vitro* (Cheng et al., 2006) or before the discovery of the EJC's anchor eIF4A3. Only the combined depletion of all EJC subunits known at the time triggered a partial block of export, and the co-depletion of Alyref and these EJC proteins is lethal in *Drosophila* cells (Gatfield and Izaurralde, 2002). Our study confirms a key functional link between the EJC and Alyref. In agreement, Alyref binding and enrichment patterns mirrored those of the EJC, whereas Chtop and Nxf1 were more alike (Figures 5A and S4A, and Figures 4B, S3A, and S5E). In addition, depletion of either Alyref or eIF4A3 increases the levels of Chtop. Both cases could be a cellular response to the loss of Alyref on the RNA, because Alyref works with Chtop to mediate TREX function (Figure 6D) (Chang et al., 2013). Because endogenous Alyref interacts poorly with EJC components (Cheng et al., 2006; Chi et al., 2013) and isn't strongly enriched in the EJC interactome (Singh et al., 2012), its proximity to the EJC is perhaps achieved through RNA-enhanced protein-protein interactions. Yet our binding assays suggest that an EJC-driven competition may contribute to the transfer of Alyref from the CBC next to the EJC (Figures S6D and S6E). We can reconcile Alyref 5′ enrichment in Figure 1F with its enrichment at EJC sites as the 5′ enrichment composed of Alyref strong binding to the 3′ end of a transcript's first exon, because this junction is spliced first and is closest to the CBC.

Surprisingly, our work has revealed the presence of the EJC on intronless RNAs. Our results are consistent with the ability of EJC subunits to bind nascent transcripts independently of pre-mRNA splicing in *Drosophila* (Choudhury et al., 2016). Moreover, eIF4A3 enhances translation of intronless mRNAs by binding to their 5′ end (Choe et al., 2014) (Figure S5F). The recruitment of splicing factors to intronless mRNAs is not unprecedented and could result from a scanning mechanism of the pre-mRNA by spliceosome components (Änkö et al., 2012; Brody et al., 2011). The whole EJC is associated with the 5′ exon before exon ligation, and eIF4A3 is the only EJC core protein to directly interact with spliceosome subunits (Zhang et al., 2017). In addition, eIF4A3 doesn't seem to recognize exons as well as Casc3 (Figures 5D and S5A) or their positions within the transcript (Figure S5D). Therefore, it could be involved in such a scanning process. Intronless RNAs are notoriously poorly exported and are subject to greater exosome-mediated degradation than regular mRNAs (Dias et al., 2010; Fan et al., 2017). Alyref and the EJC were both more enriched on multi-exons than on single-exon RNAs (Figures 5D and S5A), whereas Chtop and Nxf1 were not discriminative (Figure S5A). Given that the major mRNA export adaptor Alyref seems to help RNAs evade nuclear degradation by the exosome (Fan et al., 2017), it is plausible that Alyref's loading might be a rate-limiting step for the efficient export of intronless RNAs. In contrast, the single-exon histone mRNAs, which bind TREX subunits (but not the EJC) just as strongly as multi-exon RNAs, may have evolved more efficient ways to recruit RNA export factors (Figures 5D and S5A).

Overall, our study highlights an important role for both the CBC and the EJC in the nuclear export of RNAs in human cells and shows that upstream of their role in stimulating export, the binding of RNA export factors to their RNA targets plays a crucial role in shaping the transcriptome by influencing splicing decisions and 3′ end processing.

STAR★METHODS

Detailed methods are provided in the online version of this paper and include the following:

- KEY RESOURCES TABLE
- CONTACT FOR REAGENT AND RESOURCE SHARING
- EXPERIMENTAL MODEL AND SUBJECT DETAILS
- METHOD DETAILS
  - siRNA transfections
  - Fluorescent *In Situ* Hybridization (FISH)
  - individual nucleotide resolution CrossLinking and ImmunoPrecipitation (iCLIP)
  - Formaldehyde RNA Immuno-Precipitation (faRIP)
  - Recombinant proteins purifications
  - Co-Immunoprecipitations (co-IPs)
  - GST pulldowns
  - RNA-seq on siCtrl- and siCHTOP-treated cells
  - Cellular fractionation and RNA/Protein extraction
  - ChIP-seq
- QUANTIFICATION AND STATISTICAL ANALYSIS
  - Other datasets used
  - Gene Sets
  - iCLIP data processing
  - Identification of significantly crosslinked bases
  - Searching for enriched kmers
  - Metagene analysis
  - Enrichments over control
  - Splicing Index
  - Processing Index
  - Analysis of RNA-seq data
  - Alternate splicing of detained and retained introns
  - Identification of retained introns
  - Identification of detained introns
  - Identification of introns with increased splicing efficiency on Alyref knockdown
  - Analysis of alternative polyadenylation changes
  - Analysis of ChIP-seq data
- DATA AND SOFTWARE AVAILABILITY

SUPPLEMENTAL INFORMATION

Supplemental Information can be found online at https://doi.org/10.1016/j.molcel.2019.04.034.

ACKNOWLEDGMENTS

S.A.W. acknowledges support from Biotechnology and Biological Sciences Research Council (UK) (grants BB/N014839/1 and BB/N005430/1) and funding from the Medical Research Council via the Computational Genomics

Analysis and Training (CGAT) Programme (MC_EX_G1000902). We thank Dr. Nicholas Conrad for providing us with his mRNA-seq datasets from Control and Alyref knockdown cells. We thank the Wilson Lab members and Dr. Julie Aspen for useful discussions. We apologize to colleagues whose work could not be cited due to space constraints.

AUTHOR CONTRIBUTIONS

N.V. performed all molecular biology, iCLIP, and transcriptomic experiments with help from C.G.H. L.G. performed the ChIP-seq experiments. I.S. designed and performed all bioinformatics analyses with input from N.V., S.A.W., and D.S. N.V. and S.A.W. designed the project. N.V. and S.A.W. wrote the article with input from all authors.

DECLARATION OF INTERESTS

The authors declare no competing interests.

Received: May 9, 2018
Revised: February 25, 2019
Accepted: April 29, 2019
Published: May 16, 2019

REFERENCES

Andersen, C.B.F., Ballut, L., Johansen, J.S., Chamieh, H., Nielsen, K.H., Oliveira, C.L.P., Pedersen, J.S., Séraphin, B., Le Hir, H., and Andersen, G.R. (2006). Structure of the exon junction core complex with a trapped DEAD-box ATPase bound to RNA. Science *313*, 1968–1972.

Andersen, P.R., Domanski, M., Kristiansen, M.S., Storvall, H., Ntini, E., Verheggen, C., Schein, A., Bunkenborg, J., Poser, I., Hallais, M., et al. (2013). The human cap-binding complex is functionally connected to the nuclear RNA exosome. Nat. Struct. Mol. Biol. *20*, 1367–1376.

Änkö, M.-L., Müller-McNicoll, M., Brandl, H., Curk, T., Gorup, C., Henry, I., Ule, J., and Neugebauer, K.M. (2012). The RNA-binding landscapes of two SR proteins reveal unique functions and binding to diverse RNA classes. Genome Biol. *13*, R17.

Baejen, C., Torkler, P., Gressel, S., Essig, K., Söding, J., and Cramer, P. (2014). Transcriptome maps of mRNP biogenesis factors define pre-mRNA recognition. Mol. Cell *55*, 745–757.

Boehm, V., and Gehring, N.H. (2016). Exon Junction Complexes: Supervising the Gene Expression Assembly Line. Trends Genet. *32*, 724–735.

Boutz, P.L., Bhutkar, A., and Sharp, P.A. (2015). Detained introns are a novel, widespread class of post-transcriptionally spliced introns. Genes Dev. *29*, 63–80.

Braun, I.C., Herold, A., Rode, M., Conti, E., and Izaurralde, E. (2001). Overexpression of TAP/p15 heterodimers bypasses nuclear retention and stimulates nuclear mRNA export. J. Biol. Chem. *276*, 20536–20543.

Brody, Y., Neufeld, N., Bieberstein, N., Causse, S.Z., Böhnlein, E.-M., Neugebauer, K.M., Darzacq, X., and Shav-Tal, Y. (2011). The *in vivo* kinetics of RNA polymerase II elongation during co-transcriptional splicing. PLoS Biol. *9*, e1000573.

Broughton, J.P., and Pasquinelli, A.E. (2013). Identifying Argonaute binding sites in *Caenorhabditis elegans* using iCLIP. Methods *63*, 119–125.

Chang, C.-T., Hautbergue, G.M., Walsh, M.J., Viphakone, N., van Dijk, T.B., Philipsen, S., and Wilson, S.A. (2013). Chtop is a component of the dynamic TREX mRNA export complex. EMBO J. *32*, 473–486.

Cheng, H., Dufu, K., Lee, C.-S., Hsu, J.L., Dias, A., and Reed, R. (2006). Human mRNA export machinery recruited to the 5′ end of mRNA. Cell *127*, 1389–1400.

Chi, B., Wang, Q., Wu, G., Tan, M., Wang, L., Shi, M., Chang, X., and Cheng, H. (2013). Aly and THO are required for assembly of the human TREX complex and association of TREX components with the spliced mRNA. Nucleic Acids Res. *41*, 1294–1306.

Chi, B., Wang, K., Du, Y., Gui, B., Chang, X., Wang, L., Fan, J., Chen, S., Wu, X., Li, G., and Cheng, H. (2014). A Sub-Element in PRE enhances nuclear export of intronless mRNAs by recruiting the TREX complex via ZC3H18. Nucleic Acids Res. *42*, 7305–7318.

Choe, J., Ryu, I., Park, O.H., Park, J., Cho, H., Yoo, J.S., Chi, S.W., Kim, M.-K., Song, H.K., and Kim, Y.K. (2014). eIF4AIII enhances translation of nuclear cap-binding complex-bound mRNAs by promoting disruption of secondary structures in 5’UTR. Proc. Natl. Acad. Sci. USA *111*, E4577–E4586.

Choudhury, S.R., Singh, A.K., McLeod, T., Blanchette, M., Jang, B., Badenhorst, P., Kanhere, A., and Brogna, S. (2016). Exon junction complex proteins bind nascent transcripts independently of pre-mRNA splicing in *Drosophila melanogaster*. eLife *5*, 720.

Chu, C., Zhang, Q.C., da Rocha, S.T., Flynn, R.A., Bharadwaj, M., Calabrese, J.M., Magnuson, T., Heard, E., and Chang, H.Y. (2015). Systematic discovery of Xist RNA binding proteins. Cell *161*, 404–416.

Cohen, H.R., and Panning, B. (2007). XIST RNA exhibits nuclear retention and exhibits reduced association with the export factor TAP/NXF1. Chromosoma *116*, 373–383.

Custódio, N., Carmo-Fonseca, M., Geraghty, F., Pereira, H.S., Grosveld, F., and Antoniou, M. (1999). Inefficient processing impairs release of RNA from the site of transcription. EMBO J. *18*, 2855–2866.

Derti, A., Garrett-Engele, P., Macisaac, K.D., Stevens, R.C., Sriram, S., Chen, R., Rohl, C.A., Johnson, J.M., and Babak, T. (2012). A quantitative atlas of polyadenylation in five mammals. Genome Res. *22*, 1173–1183.

Dias, A.P., Dufu, K., Lei, H., and Reed, R. (2010). A role for TREX components in the release of spliced mRNA from nuclear speckle domains. Nat. Commun. *1*, 97.

Eckmann, C.R., Rammelt, C., and Wahle, E. (2011). Control of poly(A) tail length. Wiley Interdiscip. Rev. RNA *2*, 348–361.

Fan, J., Kuai, B., Wu, G., Wu, X., Chi, B., Wang, L., Wang, K., Shi, Z., Zhang, H., Chen, S., et al. (2017). Exosome cofactor hMTR4 competes with export adaptor ALYREF to ensure balanced nuclear RNA pools for degradation and export. EMBO J. *36*, 2870–2886.

Fanis, P., Gillemans, N., Aghajanirefah, A., Pourfarzad, F., Demmers, J., Esteghamat, F., Vadlamudi, R.K., Grosveld, F., Philipsen, S., and van Dijk, T.B. (2012). Five friends of methylated chromatin target of protein-arginine-methyltransferase[prmt]-1 (chtop), a complex linking arginine methylation to desumoylation. Mol. Cell. Proteomics *11*, 1263–1273.

Fong, N., Brannan, K., Erickson, B., Kim, H., Cortazar, M.A., Sheridan, R.M., Nguyen, T., Karp, S., and Bentley, D.L. (2015). Effects of Transcription Elongation Rate and Xrn2 Exonuclease Activity on RNA Polymerase II Termination Suggest Widespread Kinetic Competition. Mol. Cell *60*, 256–267.

Gatfield, D., and Izaurralde, E. (2002). REF1/Aly and the additional exon junction complex proteins are dispensable for nuclear mRNA export. J. Cell Biol. *159*, 579–588.

Gebhardt, A., Habjan, M., Benda, C., Meiler, A., Haas, D.A., Hein, M.Y., Mann, A., Mann, M., Habermann, B., and Pichlmair, A. (2015). mRNA export through an additional cap-binding complex consisting of NCBP1 and NCBP3. Nat. Commun. *6*, 8192.

Gromadzka, A.M., Steckelberg, A.-L., Singh, K.K., Hofmann, K., and Gehring, N.H. (2016). A short conserved motif in ALYREF directs cap- and EJC-dependent assembly of export complexes on spliced mRNAs. Nucleic Acids Res. *44*, 2348–2361.

Gruber, A.J., Schmidt, R., Gruber, A.R., Martin, G., Ghosh, S., Belmadani, M., Keller, W., and Zavolan, M. (2016). A comprehensive analysis of 3′ end sequencing data sets reveals novel polyadenylation signals and the repressive role of heterogeneous ribonucleoprotein C on cleavage and polyadenylation. Genome Res. *26*, 1145–1159.

Hauer, C., Sieber, J., Schwarzl, T., Hollerer, I., Curk, T., Alleaume, A.-M., Hentze, M.W., and Kulozik, A.E. (2016). Exon Junction Complexes Show a Distributional Bias toward Alternatively Spliced mRNAs and against mRNAs Coding for Ribosomal Proteins. Cell Rep. *16*, 1588–1603.

Hautbergue, G.M., Hung, M.-L., Golovanov, A.P., Lian, L.-Y., and Wilson, S.A. (2008). Mutually exclusive interactions drive handover of mRNA from export adaptors to TAP. Proc. Natl. Acad. Sci. USA *105*, 5154–5159.

Hautbergue, G.M., Hung, M.L., Walsh, M.J., Snijders, A.P., Chang, C., Jones, R., Ponting, C.P., Dickman, M.J., and Wilson, S.A. (2009). UIF, a new mRNA export adaptor which works together with REF/ALY, requires FACT for recruitment to mRNA. Curr. Biol. *19*, 1918–1924.

Heath, C.G., Viphakone, N., and Wilson, S.A. (2016). The role of TREX in gene expression and disease. Biochem. J. *473*, 2911–2935.

Huang, Y., Gattoni, R., Stévenin, J., and Steitz, J.A. (2003). SR splicing factors serve as adapter proteins for TAP-dependent mRNA export. Mol. Cell *11*, 837–843.

Izumikawa, K., Yoshikawa, H., Ishikawa, H., Nobe, Y., Yamauchi, Y., Philipsen, S., Simpson, R.J., Isobe, T., and Takahashi, N. (2016). Chtop (Chromatin target of Prmt1) auto-regulates its expression level via intron retention and nonsense-mediated decay of its own mRNA. Nucleic Acids Res. *44*, 9847–9859.

Jung, H., Lee, D., Lee, J., Park, D., Kim, Y.J., Park, W.-Y., Hong, D., Park, P.J., and Lee, E. (2015). Intron retention is a widespread mechanism of tumor-suppressor inactivation. Nat. Genet. *47*, 1242–1248.

Kaida, D., Berg, M.G., Younis, I., Kasim, M., Singh, L.N., Wan, L., and Dreyfuss, G. (2010). U1 snRNP protects pre-mRNAs from premature cleavage and polyadenylation. Nature *468*, 664–668.

Katahira, J., Inoue, H., Hurt, E., and Yoneda, Y. (2009). Adaptor Aly and co-adaptor Thoc5 function in the Tap-p15-mediated nuclear export of HSP70 mRNA. EMBO J. *28*, 556–567.

Katahira, J., Okuzaki, D., Inoue, H., Yoneda, Y., Maehara, K., and Ohkawa, Y. (2013). Human TREX component Thoc5 affects alternative polyadenylation site choice by recruiting mammalian cleavage factor I. Nucleic Acids Res. *41*, 7060–7072.

König, J., Zarnack, K., Rot, G., Curk, T., Kayikci, M., Zupan, B., Turner, D.J., Luscombe, N.M., and Ule, J. (2011). iCLIP—transcriptome-wide mapping of protein-RNA interactions with individual nucleotide resolution. J. Vis. Exp. *50*, 2638.

Le Hir, H., Izaurralde, E., Maquat, L.E., and Moore, M.J. (2000). The spliceosome deposits multiple proteins 20–24 nucleotides upstream of mRNA exon-exon junctions. EMBO J. *19*, 6860–6869.

Le Hir, H., Gatfield, D., Izaurralde, E., and Moore, M.J. (2001). The exon-exon junction complex provides a binding platform for factors involved in mRNA export and nonsense-mediated mRNA decay. EMBO J. *20*, 4987–4997.

Li, Y., Bor, Y.-C., Misawa, Y., Xue, Y., Rekosh, D., and Hammarskjöld, M.-L. (2006). An intron with a constitutive transport element is retained in a Tap messenger RNA. Nature *443*, 234–237.

Llorian, M., Gooding, C., Bellora, N., Hallegger, M., Buckroyd, A., Wang, X., Rajgor, D., Kayikci, M., Feltham, J., Ule, J., et al. (2016). The alternative splicing program of differentiated smooth muscle cells involves concerted non-productive splicing of post-transcriptional regulators. Nucleic Acids Res. *44*, 8933–8950.

Martin, G., Gruber, A.R., Keller, W., and Zavolan, M. (2012). Genome-wide analysis of pre-mRNA 3′ end processing reveals a decisive role of human cleavage factor I in the regulation of 3′ UTR length. Cell Rep. *1*, 753–763.

Masamha, C.P., Xia, Z., Yang, J., Albrecht, T.R., Li, M., Shyu, A.-B., Li, W., and Wagner, E.J. (2014). CFIm25 links alternative polyadenylation to glioblastoma tumour suppression. Nature *510*, 412–416.

Mauger, O., Lemoine, F., and Scheiffele, P. (2016). Targeted Intron Retention and Excision for Rapid Gene Regulation in Response to Neuronal Activity. Neuron *92*, 1266–1278.

Meola, N., Domanski, M., Karadoulama, E., Chen, Y., Gentil, C., Pultz, D., Vitting-Seerup, K., Lykke-Andersen, S., Andersen, J.S., Sandelin, A., and Jensen, T.H. (2016). Identification of a Nuclear Exosome Decay Pathway for Processed Transcripts. Mol. Cell *64*, 520–533.

Middleton, R., Gao, D., Thomas, A., Singh, B., Au, A., Wong, J.J.L., Bomane, A., Cosson, B., Eyras, E., Rasko, J.E.J., and Ritchie, W. (2017). IRFinder: assessing the impact of intron retention on mammalian gene expression. Genome Biol. *18*, 51.

Millevoi, S., Loulergue, C., Dettwiler, S., Karaa, S.Z., Keller, W., Antoniou, M., and Vagner, S. (2006). An interaction between U2AF 65 and CF I(m) links the splicing and 3′ end processing machineries. EMBO J. *25*, 4854–4864.

Müller-McNicoll, M., Botti, V., de Jesus Domingues, A.M., Brandl, H., Schwich, O.D., Steiner, M.C., Curk, T., Poser, I., Zarnack, K., and Neugebauer, K.M. (2016). SR proteins are NXF1 adaptors that link alternative RNA processing to mRNA export. Genes Dev. *30*, 553–566.

Ohno, M., Sakamoto, H., and Shimura, Y. (1987). Preferential excision of the 5′ proximal intron from mRNA precursors with two introns as mediated by the cap structure. Proc. Natl. Acad. Sci. USA *84*, 5187–5191.

Pabis, M., Neufeld, N., Steiner, M.C., Bojic, T., Shav-Tal, Y., and Neugebauer, K.M. (2013). The nuclear cap-binding complex interacts with the U4/U6·U5 tri-snRNP and promotes spliceosome assembly in mammalian cells. RNA *19*, 1054–1063.

Pandya-Jones, A., Bhatt, D.M., Lin, C.-H., Tong, A.-J., Smale, S.T., and Black, D.L. (2013). Splicing kinetics and transcript release from the chromatin compartment limit the rate of Lipid A-induced gene expression. RNA *19*, 811–827.

Qu, X., Lykke-Andersen, S., Nasser, T., Saguez, C., Bertrand, E., Jensen, T.H., and Moore, C. (2009). Assembly of an export-competent mRNP is needed for efficient release of the 3′-end processing complex after polyadenylation. Mol. Cell. Biol. *29*, 5327–5338.

Romeo, V., and Schümperli, D. (2016). Cycling in the nucleus: regulation of RNA 3′ processing and nuclear organization of replication-dependent histone genes. Curr. Opin. Cell Biol. *40*, 23–31.

Rot, G., Wang, Z., Huppertz, I., Modic, M., Lenče, T., Hallegger, M., Haberman, N., Curk, T., von Mering, C., and Ule, J. (2017). High-Resolution RNA Maps Suggest Common Principles of Splicing and Polyadenylation Regulation by TDP-43. Cell Rep. *19*, 1056–1067.

Ruepp, M.D., Aringhieri, C., Vivarelli, S., Cardinale, S., Paro, S., Schümperli, D., and Barabino, S.M. (2009). Mammalian pre-mRNA 3′ End Processing Factor CF Im68 Functions in mRNA Export. Mol. Biol. Cell. *20*, 5211–5223.

Schlackow, M., Nojima, T., Gomes, T., Dhir, A., Carmo-Fonseca, M., and Proudfoot, N.J. (2017). Distinctive Patterns of Transcription and RNA Processing for Human lincRNAs. Mol. Cell *65*, 25–38.

Shi, M., Zhang, H., Wu, X., He, Z., Wang, L., Yin, S., Tian, B., Li, G., and Cheng, H. (2017). ALYREF mainly binds to the 5′ and the 3′ regions of the mRNA *in vivo*. Nucleic Acids Res. *45*, 9640–9653.

Singh, G., Kucukural, A., Cenik, C., Leszyk, J.D., Shaffer, S.A., Weng, Z., and Moore, M.J. (2012). The cellular EJC interactome reveals higher-order mRNP structure and an EJC-SR protein nexus. Cell *151*, 750–764.

Singh, G., Pratt, G., Yeo, G.W., and Moore, M.J. (2015). The Clothes Make the mRNA: Past and Present Trends in mRNP Fashion. Annu. Rev. Biochem. *84*, 325–354.

Smith, T., Heger, A., and Sudbery, I. (2017). UMI-tools: modeling sequencing errors in Unique Molecular Identifiers to improve quantification accuracy. Genome Res. *27*, 491–499.

Strässer, K., Masuda, S., Mason, P., Pfannstiel, J., Oppizzi, M., Rodríguez-Navarro, S., Rondón, A.G., Aguilera, A., Struhl, K., Reed, R., and Hurt, E. (2002). TREX is a conserved complex coupling transcription with messenger RNA export. Nature *417*, 304–308.

Stubbs, S.H., and Conrad, N.K. (2015). Depletion of REF/Aly alters gene expression and reduces RNA polymerase II occupancy. Nucleic Acids Res. *43*, 504–519.

Stutz, F., Bachi, A., Doerks, T., Braun, I.C., Séraphin, B., Wilm, M., Bork, P., and Izaurralde, E. (2000). REF, an evolutionary conserved family of hnRNP-like proteins, interacts with TAP/Mex67p and participates in mRNA nuclear export. RNA *6*, 638–650.

Sultan, M., Amstislavskiy, V., Risch, T., Schuette, M., Dökel, S., Ralser, M., Balzereit, D., Lehrach, H., and Yaspo, M.-L. (2014). Influence of RNA extraction methods and library selection schemes on RNA-seq data. BMC Genomics *15*, 675.

Tian, B., and Manley, J.L. (2017). Alternative polyadenylation of mRNA precursors. Nat. Rev. Mol. Cell Biol. *18*, 18–30.

Tilgner, H., Knowles, D.G., Johnson, R., Davis, C.A., Chakrabortty, S., Djebali, S., Curado, J., Snyder, M., Gingeras, T.R., and Guigó, R. (2012). Deep sequencing of subcellular RNA fractions shows splicing to be predominantly co-transcriptional in the human genome but inefficient for lncRNAs. Genome Res. *22*, 1616–1625.

Viphakone, N., Hautbergue, G.M., Walsh, M., Chang, C.-T., Holland, A., Folco, E.G., Reed, R., and Wilson, S.A. (2012). TREX exposes the RNA-binding domain of Nxf1 to enable mRNA export. Nat. Commun. *3*, 1006.

Viphakone, N., Cumberbatch, M.G., Livingstone, M.J., Heath, P.R., Dickman, M.J., Catto, J.W., and Wilson, S.A. (2015). Luzp4 defines a new mRNA export pathway in cancer cells. Nucleic Acids Res. *43*, 2353–2366.

Wang, Z., Kayikci, M., Briese, M., Zarnack, K., Luscombe, N.M., Rot, G., Zupan, B., Curk, T., and Ule, J. (2010). iCLIP predicts the dual splicing effects of TIA-RNA interactions. PLoS Biol. *8*, e1000530.

Werner, M.S., and Ruthenburg, A.J. (2015). Nuclear Fractionation Reveals Thousands of Chromatin-Tethered Noncoding RNAs Adjacent to Active Genes. Cell Rep. *12*, 1089–1098.

Windhager, L., Bonfert, T., Burger, K., Ruzsics, Z., Krebs, S., Kaufmann, S., Malterer, G., L'Hernault, A., Schilhabel, M., Schreiber, S., et al. (2012). Ultrashort and progressive 4sU-tagging reveals key characteristics of RNA processing at nucleotide resolution. Genome Res. *22*, 2031–2042.

Yao, C., Biesinger, J., Wan, J., Weng, L., Xing, Y., Xie, X., and Shi, Y. (2012). Transcriptome-wide analyses of CstF64-RNA interactions in global regulation of mRNA alternative polyadenylation. Proc. Natl. Acad. Sci. USA *109*, 18773–18778.

Yoshimoto, R., Kaida, D., Furuno, M., Burroughs, A.M., Noma, S., Suzuki, H., Kawamura, Y., Hayashizaki, Y., Mayeda, A., and Yoshida, M. (2017). Global analysis of pre-mRNA subcellular localization following splicing inhibition by spliceostatin A. RNA *23*, 47–57.

Zhang, X., Yan, C., Hang, J., Finci, L.I., Lei, J., and Shi, Y. (2017). An Atomic Structure of the Human Spliceosome. Cell *169*, 918–929.

Zhu, Y., Wang, X., Forouzmand, E., Jeong, J., Qiao, F., Sowd, G.A., Engelman, A.N., Xie, X., Hertel, K.J., and Shi, Y. (2018). Molecular Mechanisms for CFIm-Mediated Regulation of mRNA Alternative Polyadenylation. Mol. Cell *69*, 62–74.

Zolotukhin, A.S., Uranishi, H., Lindtner, S., Bear, J., Pavlakis, G.N., and Felber, B.K. (2009). Nuclear export factor RBM15 facilitates the access of DBP5 to mRNA. Nucleic Acids Res. *37*, 7151–7162.

CellPress

STAR★METHODS

KEY RESOURCES TABLE

REAGENT or RESOURCE	SOURCE	IDENTIFIER
Antibodies		
anti-Alyref (11G5)	Sigma	Cat# A9979; RRID:AB_476779
anti-Ncbp1	Abcam	Cat# ab42389; RRID:AB_880708
anti-eIF4A3	Abcam	ab180573
anti-Nxf1 (53H8)	Abcam	Cat# ab50609; RRID:AB_881770
anti-Wtap	Abcam	ab195380
anti-Ddx39	Viphakone et al., 2012	N/A
anti-Tubulin	Sigma	Cat# T5168; RRID:AB_477579
anti-Chtop	Chang et al., 2013	N/A
anti-Histone 3	Abcam	Cat# ab1791; RRID:AB_302613
anti-Flag	Sigma	Cat# F3165; RRID:AB_259529
anti-Pabpn1	Abcam	Cat# ab75855; RRID:AB_1310538
anti-Zc3h14	Atlas	Cat# HPA049798; RRID:AB_2680888
anti-hnRNPA1 (9H10)	Millipore	Cat# 04-1469; RRID:AB_11213776
anti-Thoc5	Chang et al., 2013	N/A
anti-Mtr4	Abcam	ab177884
anti-phospho-Ser2 RNAPII	MBL	Cat# MABI0602; RRID:AB_2747403
anti-phospho-Ser5 RNAPII	MBL	Cat# MABI0603; RRID:AB_2728736
anti-RNAPII	MBL	Cat# MABI0601; RRID:AB_2728735
anti-Cpsf6	Bethyl	Cat# A301-356A; RRID:AB_937781
Chemicals, Peptides, and Recombinant Proteins		
pladienolide B	Santa Cruz Biotechnology	sc-391691
cordycepin	Sigma	C9137
actinomycin D	Sigma	A1410
5,6-dichlorobenzimidazole 1-β-D-ribofuranoside	Sigma	D1916
Deposited Data		
Raw experimental data	This paper	http://dx.doi.org/10.17632/jb555x5fdj.1
Raw and analyzed data	This paper	GEO: GSE113953
Experimental Models: Cell Lines		
Tetracyclin-inducible FLAG-Alyref FlpIn293	This paper	N/A
Tetracyclin-inducible FLAG-Chtop FlpIn293	This paper	N/A
Tetracyclin-inducible FLAG-Nxf1 FlpIn293	This paper	N/A
Oligonucleotides		
Primers, see Table S1	This paper	N/A
Software and Algorithms		
Salmon 0.8.2	https://github.com/COMBINE-lab/salmon	N/A
CGAT Tools	https://cgat.readthedocs.io/en/latest/cgat.html	N/A
iCLIPlib	https://www.github.com/sudlab/iCLIPlib	N/A
bowtie 1.1.2	http://bowtie-bio.sourceforge.net/index.shtml	N/A
UMI-Tools 0.5.3	https://github.com/CGATOxford/UMI-tools	N/A
Kraken tools	http://www.ebi.ac.uk/research/enright/software/kraken	N/A
STAR 2.4.2a	https://github.com/alexdobin/STAR	N/A

(*Continued on next page*)

Continued

REAGENT or RESOURCE	SOURCE	IDENTIFIER
Integrative Genomic Viewer	http://software.broadinstitute.org/software/igv/	N/A
DEXSeq	http://bioconductor.org/packages/release/bioc/html/DEXSeq.html	N/A
DaPars	https://github.com/ZhengXia/dapars	N/A
TxImport	https://github.com/mikelove/tximport	N/A
BWA MEM version 0.7.17-r1188	http://bio-bwa.sourceforge.net/	N/A

CONTACT FOR REAGENT AND RESOURCE SHARING

Further information and requests for resources and reagents should be directed to and will be fulfilled by the lead contact, Stuart Wilson (stuart.wilson@sheffield.ac.uk).

EXPERIMENTAL MODEL AND SUBJECT DETAILS

Cell lines and cell culture conditions

HEK293T, HeLa, cell lines were maintained in Dulbecco's modified Eagle medium (DMEM) with 10% fetal bovine serum (FBS). FlpIn-293 cells expressing the FLAG-tagged proteins were generated as described previously (Hautbergue et al., 2009) and maintained in DMEM with 10% FBS, 15 μg/mL Blasticidin, and 0.1 mg/mL Hygromycin.

METHOD DETAILS

siRNA transfections

Cells were transfected on the day of seeding with 7.5 nM of siRNAs (30 nM for HeLa cells) using RNAiMAX (Life technologies) according to the manufacturer's instructions. The transfection was repeated at 48 hours and the cells were harvested between 68 and 72 hours after the first transfection. The siRNAs used were: siCtrl 5′-CACCGUGAAGCUGAAGGUG-3′ (Viphakone et al., 2015), sieIF4A3 5′-AGCCACCUUCAGUAUCUCA-3′, siCHTOP 5′-GACAACCAAUUGGAUGCAUAU-3′, siCHTOP_2 5′-CAGACAGAUCCCGAAACCAAUGAUU-3′, siCHTOP_3 5′-GAUGCAUAUAUGUCGAAAA-3′, siNXF1 5′-UGAGCAUGAUUCAGAGCAA-3′, siALYREF_3UTR 5′-GAUUUAAAAACUCAUGUAAAGGUUU-3′, siCHTOP_3UTR 5′-CCACAUUGAUAAUUUAGUAAACUGA-3′.

Fluorescent *In Situ* Hybridization (FISH)

Performed as described in (Viphakone et al., 2012).

individual nucleotide resolution CrossLinking and ImmunoPrecipitation (iCLIP)

Near endogenous expression of the FLAG-tagged proteins was induced for 48h in the stable cell lines by addition of tetracycline at 5 ng/mL for Nxf1, and 10 ng/mL for Chtop, and Alyref. iCLIP was then performed exactly as described previously (König et al., 2011) but using 10 μg of anti-FLAG antibodies (Sigma) and performing the high salt washes of the immunoprecipitation part of the protocol at room temperature (22°C) on a rotating wheel for 5 minutes per wash. Additionally, the following modifications from (Broughton and Pasquinelli, 2013) were used. All nucleic acid pellets were air dry for a maximum of 10 minutes to ease resuspension. Nucleic acids centrifugations after each overnight precipitation were done at 4°C, 16000 x g for 20 minutes and 5 minutes for the EtOH 80% washes. During the cDNA gel purification step, each gel piece was transferred to a 0.5 mL microcentrifuge tube pierced with 3-4 holes made with a 19-gauge needle. The gel-containing 0.5 mL tube was itself inserted inside a 1.5 mL microfuge tube and the assembly was centrifuged at 16000 x g for 2 minutes to efficiently shred the gel piece. After circularisation of the cDNAs, the Circligase was inactivated by heating the samples for 15 minutes at 80°C. To linearize the cDNAs, the standard iCLIP cut_oligo was replaced by a cut_oligo bearing a 3′ dideoxycytosine instead of four adenosines. Libraries for each protein of interest, as well as libraries from cells expressing only the FLAG tag were prepared in triplicate. Libraries were pooled, a spike-in of 10% PhiX DNA added and distributed across 4 lanes of Illumina HiSeq 2500 and 1 lane of Illumina MiSeq sequencing by the Centre for Genomic Research (Liverpool, UK).

Formaldehyde RNA Immuno-Precipitation (faRIP)

One 6-cm dish (or 2 × 6-cm dishes for siRNA treatments) was seeded per RIP condition with 300000 cells/dish. Protein-RNA complexes were crosslinked *in vivo* 48 hours later (or 68-72 hours later for siRNA treatments) by incubating the cells with 3 mL of PBS-Formaldehyde (0.1%). 100 μL of protein G-Dynabeads were prepared by initial washing with 3 × 1 mL RIP lysis buffer (50 mM HEPES-HCl pH 7.5, 150 mM NaCl, 10% glycerol 1% NP-40, 0.1% SDS, and 0.5% sodium deoxycholate) before being blocked

and loaded with the relevant antibody (4 μg diluted in 0.3 mL of RIP lysis buffer + 1% BSA w/v final ; control RIP antibody was anti-FLAG, Sigma) for 1 hour at room temperature. The beads were then washed with 3 × 1 mL RIP lysis buffer and left on ice until further use. Each cell pellet was lysed in 400 μL RIP lysis buffer supplemented with 1 mM DTT, protease inhibitors (SigmaFAST, Sigma), 2 μL of RNase inhibitors (Ribosafe, Bioline) and 2 μL/mL of Turbo DNase (Ambion). Samples were then sonicated using a Bioruptor (High, 8 x [30 s-ON/30 s-OFF]) to generate fragments of ~350 nts, and cleared by centrifugation (16100 x g, 10 minutes, 4°C). 300 μL of each sample were incubated with the prepared Dynabeads for 2 hours at 4°C and 30 μL of lysate were kept as an input (10%). Following incubation, the beads were washed with 2 × 1 mL RIP lysis buffer, 2 × 1 mL high salt RIP lysis buffer (adjusted to 500 mM NaCl, 5 minutes each on ice), and again 2 × 1 mL RIP lysis buffer. Crosslinks reversal and elutions were performed by adjusting inputs and washed beads to 100 μL with reverse-crosslinking buffer (final concentrations: PBS 1X, 2% N-lauroyl sarcosine, 10 mM EDTA, 5 mM DTT, 1.9 mg/mL proteinase K (Roche)) and shaking them at 1100 rpm for 1 hour at 42°C followed by 1 hour at 55°C. The RNA content of the resulting eluates and inputs were extracted using TRIzol (Life Technologies), following the manufacturer's instructions. All RNA samples were then DNase-treated (Turbo DNase, Ambion), phenol/chloroform extracted, ethanol precipitated, and resuspended in RNases-free water (Sigma). The whole content of RNAs obtained from the immunoprecipitations and inputs were used for cDNA synthesis and qPCR analysis. Oligonucleotides for qPCR analysis are listed in Table S1.

Recombinant proteins purifications

eIF4A3-6His, Ncbp1-6His and [Magoh:Rbm8a-6His] were produced in BL21-RP *E.coli* cells, and purified using TALON cobalt beads according to the manufacturer's instructions. They were then concentrated and buffer exchanged five times against RB100 buffer using Vivaspin concentrators (Sartorius). The heterodimer Magoh:Rbm8a-6His and the helicase eIF4A3-6His were incubated at a 1:1 molar ratio in RB100 supplemented with 200 μM ATP[β,γ-NH] at room temperature for at least 30 minutes with rotation before being used in competition experiments (see below).

Co-Immunoprecipitations (co-IPs)

HEK293T cells were seeded at 60% confluency in 10-cm dishes (15-cm dishes for the co-IPs with RNAPII phosphoforms). Where indicated, the cells were treated with either actinomycin D (5 μg/mL, Sigma), 5,6-dichlorobenzimidazole 1-β-D-ribofuranoside (DRB, 100 μM, Sigma), pladienolide B (1 μM, Santa Cruz Biotechnology), cordycepin (25 μM, Sigma), or DMSO (as mock-treated negative control) for 3 hours prior to harvesting. 4 μg of the indicated antibodies (or anti-FLAG for control IPs) were incubated with 100 μL of protein G Dynabeads for 1 hour at room temperature in 300 μL of IP lysis buffer (50 mM HEPES-NaOH pH 7.5, 100 mM NaCl, 1 mM EDTA pH 8, 0.1% Triton X-100, 10% Glycerol) supplemented with 1% BSA. The cells were briefly washed in ice-cold PBS and lysed in ice-cold IP lysis buffer supplemented with 1 mM DTT, Turbo DNase (4 U/mL), protease inhibitors (SigmaFast), and RNase A (10 μg/mL). Equivalent amounts of cleared protein extracts were incubated with the prepared antibody-bound protein G Dynabeads for 2 hours at 4°C. The beads were then washed three times with 1 mL of IP lysis buffer containing RNase A (10 μg/mL) and bound proteins were eluted by an acid shock using 1 M Arginine-HCl (pH 3.5) and neutralised to pH 7.5 using 1.5 M Tris-HCl pH 8.8. 0.1%–0.2% of inputs and 16%–30% of eluates were subsequently analyzed by SDS-PAGE and western blotting using the indicated antibodies. The co-IPs reported in Figure S6G were performed with the following variations: 15-cm dishes of cells were used (80% confluency), RNase A was replaced by 40 U/mL RNase inhibitors (Ribosafe, Bioline), the IP lysis buffer washes were followed by three washes with 1 mL of RB100 Buffer (25 mM HEPES pH 7.5, 100 mM KOAc, 10 mM $MgAc_2$, 1 mM DTT, 0.05% Triton X-100, 10% glycerol), and binding competitions were initiated by adding 1.7 μM of recombinant EJC or Bovine Serum Albumin (BSA) in a final volume of 500 μL of RB100 buffer with rotation for 2 hours at 4°C. Finally, the beads were then washed three times with 1 mL of RB100 buffer and bound proteins were eluted and analyzed as described above.

GST pulldowns

GST and GST-Alyref were expressed in BL21-RP *E.coli* cells and purified using Glutathione Sepharose according to the manufacturer's instructions (GE Healthcare) using PBS-T buffer (1 × PBS, 0.1% Tween, RNase A 10 μg/mL). The beads and their bound GST fusions were then equilibrated in RB100 buffer. Binding reactions were performed with 100 ng of Ncbp1 in 0.4 mL of RB100 buffer with RNase A 10 μg/mL for 40 min at 4°C with rotation. The beads were then washed three times with 1 mL of the same buffer. Competitions were triggered with increasing concentrations of recombinant eIF4A3-6His or Bovine Serum Albumin (4.8 nM, 48 nM, 192 nM, 384 nM) and incubation in same conditions. The beads were then washed as above. Finally, the bound proteins were eluted with GSH Elution buffer (50 mM Tris–HCl (pH 8.2), 40 mM GSH reduced, 100 mM KOAc) and analyzed by SDS-PAGE.

RNA-seq on siCtrl- and siCHTOP-treated cells

600 000 cells were seeded in a 10-cm dish and subjected to siRNA-mediated knockdown (see above). Total RNA extraction was performed using TRIzol following the manufacturer's instructions. Samples were resuspended in 50 μL of RNase-free water (Sigma), DNase-treated with 4 U of Turbo DNase (Ambion) for 1 hour at 37°C, phenol-extracted, ethanol-precipitated overnight at −20°C and resuspended in 40 μL of RNase-free water. mRNA enrichment, cDNA generation, strand-specific library preparation and sequencing were performed using standard Illumina protocols by Novogene (Beijing, China). At least 20 millions read pairs were generated.

Cellular fractionation and RNA/Protein extraction

Nuclear and cytoplasmic proteins and RNAs were extracted from cells as described previously (Stubbs and Conrad, 2015) with the following two modifications: first Ribosafe (0.1 U/mL) was used in place of RNasin (0.04 U/mL), and second, after cell lysis, the nuclei were additionally washed twice with "Buffer I" (10 mM Tris pH 8.0, 0.32 M Sucrose, 3 mM $CaCl_2$, 2 mM $MgCl_2$, 0.1 mM EDTA, 1 mM DTT, 10% glycerol). To extract nucleoplasmic and chromatin-associated proteins, the purified nuclei were resuspended in 350 μL of NRB buffer (20 mM HEPES pH 7.5, 50% Glycerol, 75 mM NaCl, 1 mM DTT, protease inhibitors SigmaFast) and then an equal volume of NUN buffer (20 mM HEPES, 300 mM NaCl, 1 M Urea, 1% NP-40 Substitute, 10 mM $MgCl_2$, 1 mM DTT) was added and incubated 5 minutes on ice, then centrifuged (1,200 x g, 5 minutes, 4°C). The supernatant was transferred to another tube and corresponded to the soluble nucleoplasmic extract. The crude chromatin pellet was resuspended in 1 mL of Buffer A (10 mM HEPES pH 7.5, 10 mM KCl, 10% glycerol, 4 mM $MgCl_2$, 1 mM DTT, protease inhibitors SigmaFast) to wash, transferred to another 1.5 mL tube, and centrifuged (1,200 x g, 5 minutes, 4°C). The resulting purified chromatin pellets were resuspended in 100 μL of RIPA buffer (50 mM HEPES pH 7.5, 150 mM NaCl, 1 mM DTT, 10% glycerol, 1% NP-40, 0.1% SDS, 0.5% sodium deoxycholate + protease inhibitors SigmaFast) and treated with 500 U of Benzonase at room temperature for 45 minutes to release the chromatin-associated proteins. The digested chromatin was cleared by centrifugation (16100 x g, 10 minutes, 4°C) and the supernatant was analyzed by SDS-PAGE and western blot with the indicated antibodies.

ChIP-seq

Three 15-cm dishes were seeded per ChIP condition with 5×10^6 cells/dish. Near endogenous expression of the FLAG-tagged proteins was induced for 48 hours in the stable cell lines by addition of tetracycline at 5 ng/mL for Nxf1, and 10 ng/mL for Chtop, and Alyref. Protein-DNA complexes were cross-linked *in vivo* by incubating the cells with 20 mL PBS-formaldehyde (1%). Cell pellets were lysed in ChIP Lysis Buffer 1 (50 mM HEPES-NaOH pH7.5, 140 mM NaCl, 1 mM EDTA, 10% glycerol, 0.5% NP40, 0.25% Triton X-100, protease inhibitors SigmaFAST) and rotated at 4°C for 5 minutes. Nuclei were then pelleted via centrifugation (3000 x g, 5 minutes at 4°C), and resuspended in ChIP Buffer 2 (10 mM Tris-HCl pH 7.3, 200 mM NaCl, 0.5 mM EGTA, 1 mM EDTA, protease inhibitors SigmaFAST), before rotation at room temperature for 10 mins. Nuclei were pelleted via centrifugation (1500 x g, 5 minutes at 4°C) and resuspended in ChIP Lysis Buffer 3 (10 mM Tris-HCl pH 7.3, 200 mM NaCl, 0.5 mM EGTA, 1 mM EDTA, 0.1% Na-deoxycholate, 0.5% N-lauroylsarcosine, protease inhibitors SigmaFAST). Samples were sonicated using a Bioruptor (High, 20 x [30 s-ON/30 s-OFF]) to generate chromatin fragments of 250-300 nts, and cleared by centrifugation (16100 x g, 15 minutes, 4°C). Lysate concentrations were measured by Bradford assay, and equal concentrations of chromatin were incorporated into the IPs. IPs were carried out overnight at 4°C using 5 μg of FLAG antibody (Sigma). 100 μL blocked protein-G Dynabeads were then added to the samples and incubated for 2 hours at 4°C. Following incubation, beads were washed with 4 × 0.5 mL RIPA Wash Buffer (50 mM HEPES-NaOH pH 7.5, 500 mM LiCl, 1 mM EDTA, 1% NP40, 0.7% Na-deoxycholate, 0.1% N-lauroylsarcosine) and once with 0.5 mL ChIP Final Wash Buffer (10 mM Tris-HCl pH7.3, 1 mM EDTA, 50 mM NaCl). Complexes were eluted by adding 200 μL of ChIP Elution buffer (50 mM Tris-HCl pH8.0, 10 mM EDTA, 1% SDS) and incubated at 65°C for 30 minutes. NaCl was added to a final concentration of 200 mM and cross-links were reversed overnight at 65°C. Samples were then treated with RNase A (0.2 mg/mL) for 2 hours at 37°C, followed by proteinase K (0.2 mg/mL) for 2 hours at 55°C. DNA was purified via phenol-chloroform extraction and ethanol precipitation, and then resuspended in water. Library preparation and sequencing were performed using the ChIP-seq library protocol of Novogene (Beijing, China).

QUANTIFICATION AND STATISTICAL ANALYSIS

Other datasets used

eIF4A3 and Casc3 iCLIP data were downloaded from ENA accession ERA551949 (Hauer et al., 2016). Total nuclear and cytoplasmic ribosome-depleted RNA-seq from HEK293 cells was downloaded from ENA accession PRJEB4197 (Sultan et al., 2014). Nuclear mRNA-seq from HEK293 was downloaded from GEO accession GSE111878 (manuscript submitted). HEK293 A-seq was downloaded from GEO accession GSM909242 (Martin et al., 2012). Nuclear and cytoplasmic mRNA-seq data from Control and Alyref knockdown HEK293 cells were the kind gift of Nicolas Conrad (Stubbs and Conrad, 2015) and were provided as pre-mapped alignments. Endogenous Alyref iCLIP data were from GSE99069 (Shi et al., 2017).

Gene Sets

Throughout the study two gene sets were used. The 'reference' set was obtained from Ensembl 75. The 'expressed' set was derived using transcripts with mean TPM > 1 as measured by Salmon 0.8.2 (https://github.com/COMBINE-lab/salmon) using total nuclear RNA-seq. We excluded genes on the mitochondrial chromosome. To annotate different parts of genes, we first divided genes from the reference set into the minimum number of non-overlapping chunks such that any isoform of the gene could be constructed from a combination of these chunks. This was performed using the 'genes-to-unique-chunks' mode of the 'gtf2gtf' tool from CGAT (https://cgat.readthedocs.io/en/latest/cgat.html). For each chunk, we then counted the number of exons and introns it overlapped with from both the reference and expressed gene sets using 'bedtools intersect'. We excluded any chunks that overlapped with

more than one gene. We defined introns as chunks that overlapped with at least one intron from the expressed transcript set, but no exons from any isoform; and exons as chunks that overlapped with at least one exon from the expressed transcript set, but no introns from any isoform.

iCLIP data processing

iCLIP sequencing data was processed using pipeline_iCLIP distributed as part of iCLIPlib (https://www.github.com/sudlab/iCLIPlib). For data generated for this study, we removed spiked in phiX sequence, by mapping reads against the phiX genome using bowtie 1.1.2 (http://bowtie-bio.sourceforge.net/index.shtml) with the settings '-v 2 --best-strata -a'. UMI sequences were extracted using UMI-Tools 0.5.3 (Smith et al., 2017) leaving the sample barcode on the read sequence. Reads were then simultaneously trimmed and demultiplex using Reaper from the Kraken tools (http://www.ebi.ac.uk/research/enright/software/kraken). Only reads longer than 15 nts were retained. The remaining reads were then mapped against the hg19 genome sequence using STAR 2.4.2a (https://github.com/alexdobin/STAR) and a junction database built from Ensembl 75. The options to STAR were '--outFilterMultimapNmax 1 --outFilterType BySJout --outFilterMismatchNoverLmax 0.2 --outFilterScoreMin 0.8 --alignSJDBoverhangMin 1'. Thus, multimapping reads, reads with greater than 20% mismatches or an alignment score less than 0.8 were discarded. Only splice junctions present in Ensembl 75 were allowed, but only an overhang of 1 nucleotide was required for splicing at an annotated junction. This prevents spuriously overhanging sequences from affecting calculations of splicing ratio (see below). Reads from the same sample sequenced on different lanes were then merged and PCR duplicates removed accounting for sequencing and PCR errors using UMI-Tools 'dedup'. For each read the cross-linked base was either the 5′ most deletion in the read, or the base immediately 5′ to the read end if no deletion was present. For eIF4A3 and Casc3 iCLIP datasets, obtained reads were already filtered, trimmed and demultiplexed and entered the above process at the mapping stage. As recommended, we used the center of each read to represent the cross-link site when no deletion was present (Hauer et al., 2016). We treated each replicate separately, and also report results from a 'union' set generated by merging iCLIP tags from all replicates for a factor. All iCLIP track visualizations were prepared using the Integrative Genomic Viewer (http://software.broadinstitute.org/software/igv/).

Identification of significantly crosslinked bases

As our analyses suggested that components of the TREX complex bind in a broad manner and not to specific "binding sites," most analyses are conducted using all iCLIP reads. Where specifically noted, significant bases were identified using the procedure outlined in (Wang et al., 2010) and implemented in the 'significant_bases_by_randomization' script from iCLIPlib. For each gene we merged all overlapping exons and then divided the gene into a single exonic region and a separate region for each intron. Within each region the height of an individual base is the number of all crosslink bases within 15 nts. For each region we calculate the empirical distribution of base heights, such that P_h is the fraction of bases in a region with $height > h$. To calculate an FDR, we randomize the location of crosslinks within the region and calculate P_h for the randomized profile. This procedure is repeated 100 times, and we then calculate the FDR for a base with height $\geq h$ as $FDR(h) = (\mu_h + \sigma_h)/P_h$ where μ_h and σ_h are the mean and standard deviation of P_h from the randomizations. We selected bases with FDR < 0.1 as significant. Clusters of significant bases were determined by merging significant bases within 15 nts of each other.

Searching for enriched kmers

Enrichment for kmer motifs was conducted using the z-score approach as used in (Wang et al., 2010) as implemented by 'iCLIP_kmer_enrichment' from iCLIPlib. All exons for each gene were merged. We then calculated the height of each base in a gene as the number of crosslinks within 15nt of the position. For each possible sequence s of length k, we then calculated its frequency as $f_s = \sum_{i \in P} h_i$ where P is the set of locations at which a match to the kmer begins (across the whole transcriptome) and h_i is the height at location i. We then calculate $z_s = (f_s - \mu_s)/\sigma_s$ where μ_s and σ_s are the mean and standard deviation of f_s from 100 randomizations of the crosslink positions, where randomizations take place within genes. We calculated z-score for k = 6 and 7 for each replicate of each factor and for the union of all replicates, using both all iCLIP tags and only significantly crosslinked bases, for both all exonic sequence and for 3′ UTRs only. We noted that z-scores between pulldowns were correlated with z-scores from the controls and both were correlated with the uracil content of the kmer. To account for possible crosslinking preferences, we calculated the distance of the z-score for the kmer in the test protein from a linear regression line of z scores from the test protein against z-score from the control iCLIP.

Metagene analysis

Metagene profiles of iCLIP data across genes were obtained using 'iCLIP_bam2geneprofile' from iCLIPlib. Expressed (see above) protein coding transcripts were divided into a fixed number of bins and iCLIP tags in each bin summed. Flanking regions were scaled so that bins in the flanks were the same size as the exonic bins. Profiles over each gene were normalized to the sum across all regions (usually upstream, exons, downstream) for that gene. Genes with zero tags were excluded. The final profile was then calculated by summing across all genes. For profiles with separate regions for UTRs and CDS, each region from each expressed protein coding transcript was divided into a number of bins (20, 100 and 70 for 5′ UTR, CDS and 3′ UTR respectively). Tag counts in each bin were calculated and normalized to the size of the bin for that region and that transcript. Counts for each transcript were then normalized to

the sum of all normalized bin counts for that transcript. We repeated this process for total nuclear RNA-seq and divided each iCLIP profile by the RNA-seq profile. This controls for miss-annotation of the start of 5′ UTRs and the end of 3′ UTRs. For exon-exon junction profiles, we first excluded the first and last junction in each transcript and then excluded junctions where the exons on either side of the junction were less than 100 nts in length. For each of the remaining junctions we counted the number of iCLIP tags at each position within 100 nts of the junction (in transcript coordinates) and normalized to the total number of iCLIP tags within 100 nts either side of the junction. Profiles were then calculated by summing over all junctions and normalized to the sum of the profile.

Enrichments over control

We counted iCLIP tags in each gene and each 'transcript chunk' (see above) using 'count_clip_sites' from iCLIPlib. The gene biotype was that assigned by Ensembl (see https://www.gencodegenes.org/ for definition of each biotype). Enrichments over control and confidence intervals were calculated using the 'boot_ci' function from iCLIPlib. For gene biotypes, enrichment was calculated by summing tag counts for genes in a category (e.g., protein coding genes) and dividing by the sum of tag counts for the control. In order to measure our uncertainty, we took 1000 bootstrap samples of genes and for each bootstrap calculated the ratio of pulldown tag counts to control tag counts. Confidence intervals encompass 95% of the values from these samples. The same process was used for the calculation of enrichment over control for other categories, with the exception that bootstrap samples of exons were taken where categories were categories of exons rather than transcripts (e.g., first, middle, last exons or short versus long exons). For nuclear/cytoplasmic lncRNAs, we took transcripts assigned the lincRNA biotype. To assign transcripts to nuclear or cytoplasmic categories, we generated read counts from the nuclear and cytoplasmic total RNA seq (see above). We assigned a gene to the nuclear category if the expression was significantly higher in the nucleus as measured by DESeq2 with a 5% FDR cutoff. Enrichments over control and confidence interval was calculated as above for biotypes.

Splicing Index

To calculate splicing index, we first collected reads that overlapped introns. We then put reads into one of 4 categories: Spliced (S) reads were those that overlapped both exons flanking the intron and contained a splice at exactly the annotated coordinates; Exon-Intron (EI) reads had at least 3 nts aligned to the 3′ end of the 5′ exon and at least 3 nts aligned to the 5′ end of the intron; Intron-Exon (IE) reads had at least 3 nts aligned to the 3′ end of the intron and at least 3 nts aligned to the 5′ end of the 3′ exon; and "other" reads. The splicing index (SI) was calculated as:

$$SI = \log_2 \sum_{j \in J} \frac{2 \times S_j}{EI_j + IE_j}$$

where J is the set of all annotated junctions and Sj, EI_j and IE_j is the count of Spliced, Intron-Exon and Exon-Intron reads at junction j respectively.

Processing Index

To calculate the processing index, we obtained HEK293 A-seq data (Martin et al., 2012) and used it to select the 3′ most poly-A site overlapping the expressed transcripts from each gene. For each gene g in the set, we counted the number of iCLIP tags in a 50 nts window upstream (u_g) and downstream (d_g) of each poly-A site. As the signal upstream of the poly-A site originates from both processed and unprocessed transcripts, while that downstream originates only from unprocessed, we estimate counts from processed transcripts as $u_g - d_g$. Thus, the processing index is:

$$PI = \log_2 \frac{\sum_{g \in G} d_g}{\sum_{g \in G} u_g - d_g}$$

Analysis of RNA-seq data

Reads for mRNA-seq of siControl and siChtop transfected cells were adaptor and quality trimmed, removing bases with a Q < 15 from the 5′ end of the read, Q < 20 from the 3′ end of the read and also if the average quality in a 4 nts window falls below 15. Trimmed reads greater than 36 nts were retained.

These reads and ribosome depleted nuclear and cytoplasmic total RNA from PRJEB4197 were mapped against the hg19 genome sequence using STAR 2.4.2a and a junction database built from Ensembl 75. The options to STAR were '--outFilterType BySJout --outFilterMismatchNmax 5'. All RNA-seq track visualizations were prepared using the Integrative Genomic Viewer (http://software.broadinstitute.org/software/igv/).

Alternate splicing of detained and retained introns

We defined two categories of potentially inefficiently spliced introns: Retained and Detained. "Retained introns" are isoforms of a gene where an intron is retained compared to another isoform. Detained introns are as defined by (Boutz et al., 2015) and are introns with higher than usual RNA-seq signal in a region not defined as an exon in any isoform of a gene.

Identification of retained introns

To identify retained introns, we took all the isoforms of a gene and compared them pairwise. Where all introns in transcript A are present in transcript B, but transcript B contains introns not present in transcript A that are contained between the start and end of transcript A, we call these "retained introns."

Identification of detained introns

To identify "detained" introns, we applied the method of (Boutz et al., 2015). We compared intronic read counts from nuclear mRNA-seq to counts from *in silico* constructed null repeats. We first counted reads in each transcript chunk that overlaps no exons (i.e., constitutive introns), requiring a 10 nts overlap and disallowing multimapping reads. We then used DESeq2 to calculate library size normalized counts for each of the three replicates. We then used the normalized total of intronic reads from each gene to generate one null replicate for each of the real replicates. For each intron, we calculated a weight based on its effective length:

$$w_j = \frac{\sqrt{L_j^{eff}}}{\sum_{i \in I} \sqrt{L_i^{eff}}}$$

Where I is the set of introns in a gene and L_j^{eff} is the effective length of intron j and is equivalent to the number of positions which a valid read alignment could begin from and still be counted as coming from that intron. It is calculated as:

$$L_j^{eff} = L_j^G + L^{read} - 20 - M_j$$

Where L_j^G is the genomic length of the intron, L^{read} is read length, 20 is twice the amount of overlap required for a read to count and M_j is the number of positions in the intron that would lead to a multimapping read as determined from the 100-mer alignability track downloaded from http://genome.ucsc.edu/cgi-bin/hgFileUi?db=hg19&g=wgEncodeMapability. Introns with a weight of 0 were excluded from further consideration. For each replicate, we now distributed the sum of normalized intronic reads between the introns of a gene using the weights. If $C_{i,r}$ is the normalized counts of reads in intron i in replicate r, then the null counts are:

$$N_{i,r} = w_i \times \sum_{j \in I} C_{j,r}$$

We repeated this process for all three replicates of RNA-seq data and then used DESeq2 to identify introns with a significantly higher than expected read count using an FDR threshold of 1% and only taking those with at least 4 times the expected count.

Identification of introns with increased splicing efficiency on Alyref knockdown

To identify introns with increased splicing efficiency using nuclear mRNA-seq of Control and Alyref knockdown cells, we first counted reads that mapped to each 'chunk' of a gene (see above) using 'featureCounts'; counting only those reads that overlapped chunks by at least 10 nts, and keeping multi-mapped reads (but only the primary alignment), that is with the options ' -O --primary -M --minOverlap 10 -f'. We then applied DEXSeq (http://bioconductor.org/packages/release/bioc/html/DEXSeq.html) to these counts to identify differential exon (or intron) usage, selecting chunks using a 10% FDR threshold and at least a 1.5 fold increase compared to the rest of the gene. To calculate the Alyref density on each transcript chunk, we took the $\log_2$ of the number of Alyref iCLIP tags from all replicates divided by the genomic size of the chunk. Chunks of less than 10 bp were excluded from further analysis. We then divided the range of Alyref binding densities into three equally sized bins (Low, Medium and High). We compared the differentially spliced regions to our annotations of retained and detained introns, described above, and calculated the fraction of retained, detained and other introns whose levels had increased compared to other regions of the gene.

Analysis of alternative polyadenylation changes

To determine genes with a change in poly-A site usage upon Chtop knockdown, we converted mRNA-seq reads from siControl and siCHTOP transfected HEK293T cells into read depth and used the DaPars program (https://github.com/ZhengXia/dapars) to select genes with an absolute change in Percentage of Distal pA site Usage Index (PDUI) of greater than 0.25, with an FDR threshold of 5%. To calculate change in weighted transcript length, we quantified transcript expression using Salmon v0.8.2. We then used the TxImport package (https://bioconductor.org/packages/3.4/bioc/html/tximport.html) to calculate the expression weighted transcript length for each gene. Change in length for each gene was calculated as the $\log_2$ ratio of mean length in siChtop transfected cells divided by mean length in siControl transfected cells.

Analysis of ChIP-seq data

Reads were adaptor and quality trimmed, removing bases with a $Q < 15$ from the 5′ end of the read, $Q < 20$ from the 3′ end of the read and also if the average quality in a 4 nts window falls below 15. Trimmed reads greater than 36 nts were retained. These reads were then mapped to hg19 using BWA MEM version 0.7.17-r1188 (http://bio-bwa.sourceforge.net/) with the options '-M --k 25'. The resulting alignments were filtered to remove reads mapping to more than one location, other non-primary alignments, alignments that are not "proper-paired" and alignments with an alignment score < 30. PCR duplicates were removed. Meta-gene profiles

were constructed using 'bam2geneprofile' from 'CGAT' (https://cgat.readthedocs.io/en/latest/cgat.html) against a gene set generated by removing from the expressed set defined above, any genes overlapping or within 1.25 kb of another gene. Reads were shifted 40 bp inward toward the fragment center, and resulting profiles were area normalized. Code for this process is available at https://www.github.com/sudlab/pipeline_metagenes, with the version tagged "viphakone_et_al." To generate the final metagenes, the profile from each pulldown was normalized to the input.

DATA AND SOFTWARE AVAILABILITY

Raw and processed sequencing data have been deposited in the GEO archive with accession GSE113953.

The iCLIPlib software is available from https://www.github.com/sudlab/iCLIPlib. The precise version used in this manuscript is tagged "Viphakone_et_al_release." This includes pipeline_iCLIP used for basic iCLIP processing. Code used for further analysis and generation of figures is available at https://www.github.com/sudlab/Viphakone_et_al.

Molecular Cell, Volume *75*

Supplemental Information

Co-transcriptional Loading of RNA Export Factors Shapes the Human Transcriptome

Nicolas Viphakone, Ian Sudbery, Llywelyn Griffith, Catherine G. Heath, David Sims, and Stuart A. Wilson

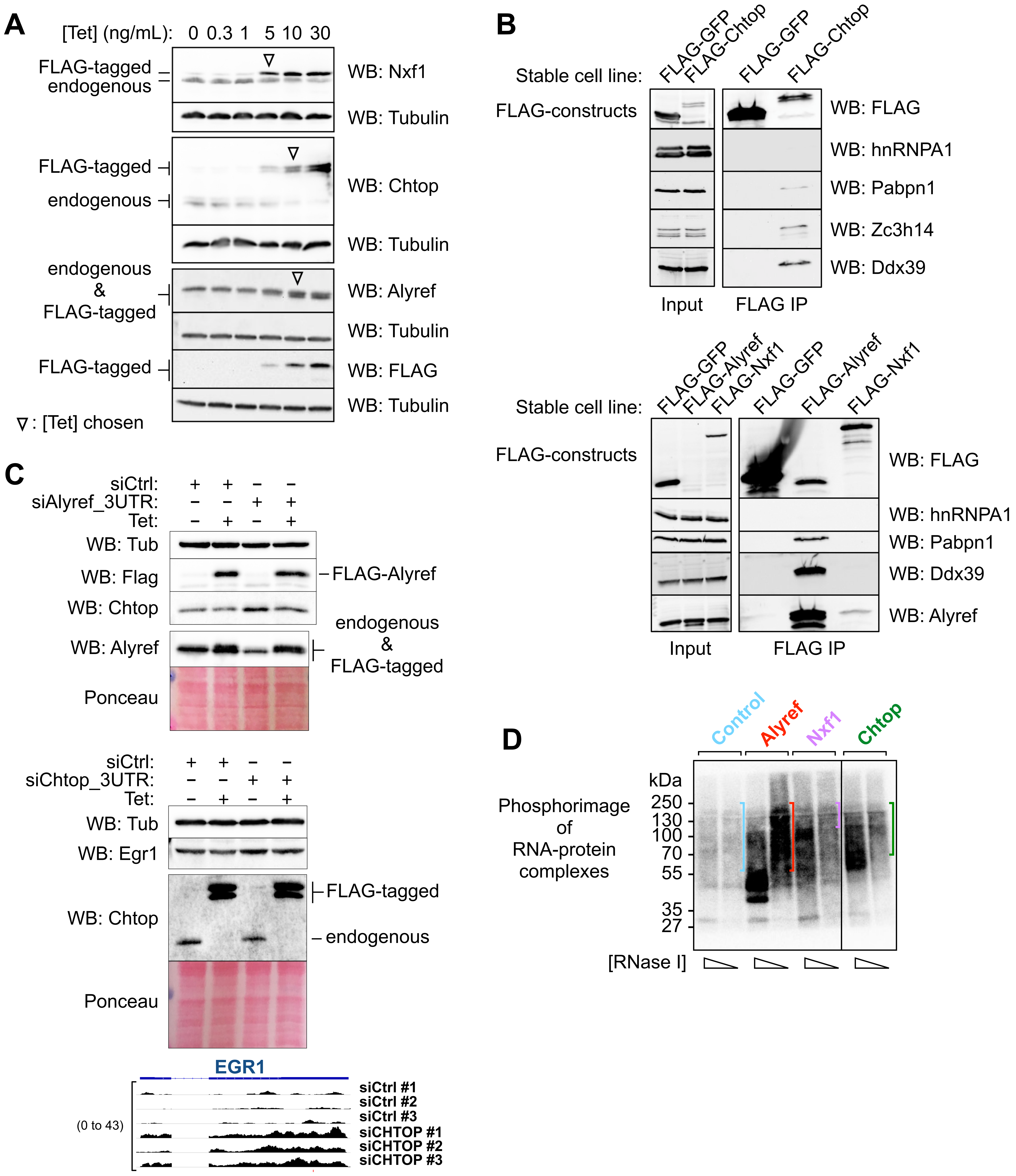

Figure S1. Validations of the FLAG-tagged constructs used for iCLIP. Related to Figure 1. A. Determination of tetracycline concentrations used for iCLIP from cell lines expressing tetracycline-inducible FLAG-tagged Alyref, Chtop, and Nxf1. Open arrowheads indicate the concentration of tetracycline used. **B.** Known interactors co-immunoprecipitate with FLAG-tagged constructs Alyref, Chtop, and Nxf1. hnRNPA1 serves as a negative control (Chang et al., EMBO 2012). **C.** Tetracyline-induced expression of Flag-tagged proteins functionally complements the loss of endogenous proteins by correcting Chtop up-regulation triggered by Alyref knockdown previously described in (Chang et al., EMBO 2012) and Egr1 up-regulation triggered by Chtop knockdown. **D.** Examples of RNA-protein crosslinks obtained during the iCLIP procedure, with coloured brackets indicating range of sizes selected.

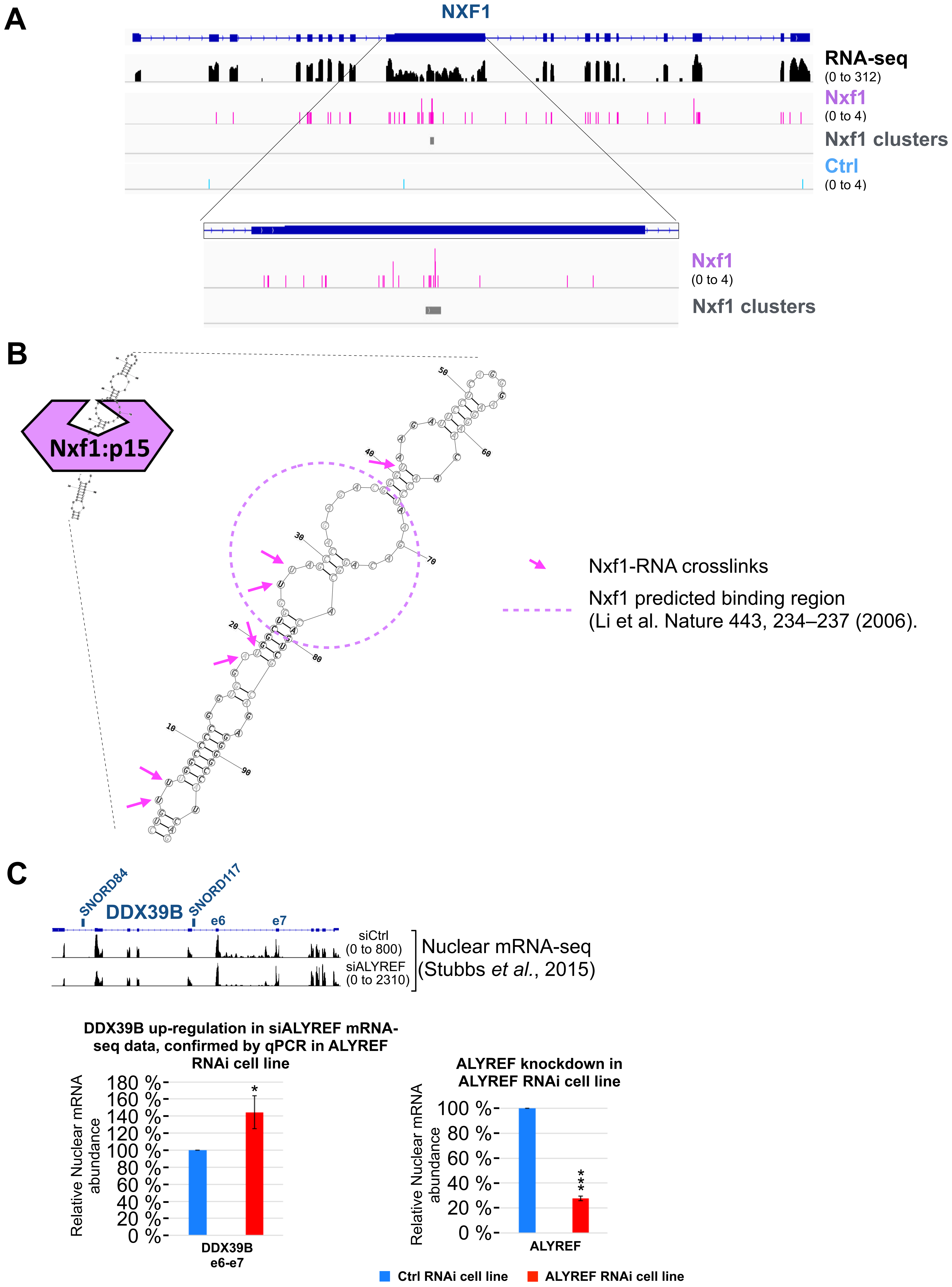

Figure S2. Nxf1 binding to an RNA secondary structure located within intron 10 of its own pre-mRNA, related to Figure 3. Characterisation of Nxf1 binding to intron 10 of its own transcript. **A.** Nxf1 iCLIP confirms the existence of Nxf1 binding cluster within intron 10 of the NXF1 transcript. **B.** Nxf1 crosslinks positions within the CTE-like RNA structure of its intron 10, with indicated predicted Nxf1 binding region on that transcript. **C.** ALYREF knockdown using a different RNAi system and target sequence (Viphakone et al., 2012) reproduces the splicing effect seen in the mRNA-seq data (Stubbs et al., 2015) that we used in our study.

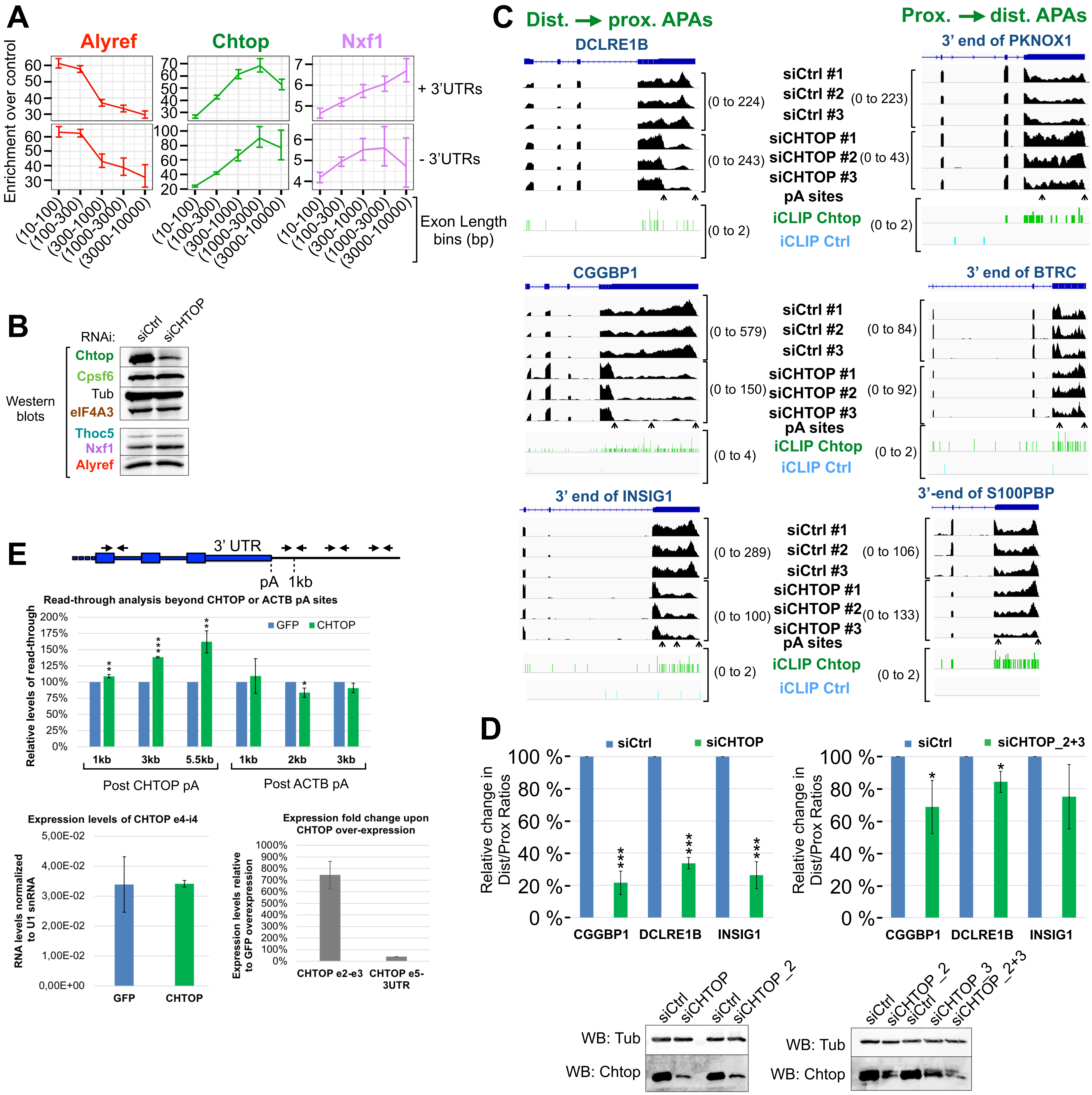

Figure S3. APA and transcription termination regulation by CHTOP. Related to Figure 4. A. Binding enrichment of export factors relative to increasing exon length with 3' UTRs included or not in the analysis. **B.** Western blot analysis of samples used for RNA-seq. 293T cells were subjected to siCHTOP for 72h. **C.** All replicates from Figure 4G and additional examples of Proximal-Distal and Distal-Proximal APA changes. "#number" = replicates. pA sites positions used were from HEK293 A-seq (Martin et al., 2012). **D.** Some APA changes caused by CHTOP knockdown and presented in Figure S3C are validated by qPCR using the siCHTOP siRNA or a less efficient combination of two other siRNAs (siCHTOP_2 and siCHTOP_3). Knockdown efficiencies were assessed by WB analysis. Means of three biological replicates +/- SD. *P< 0.05, **P< 0.01, ***P < 0.001 (t-test). **E.** Read-through transcription beyond CHTOP and ACTB genes was assessed using GFP- or CHTOP-over-expressing stable cell lines. Read-through was normalised to pre-mRNA levels (region e4-i4 in case of CHTOP) and expressed as fold change over signal from GFP-overexpressing cells. Also shown is the down-regulation of endogenous CHTOP mRNA (primers pair "e5-3UTR") upon CHTOP over-expression (primers pair "e2-e3"). Means of three independent experiments +/- SD. *P< 0.05, **P< 0.01, ***P < 0.001 (t-test).

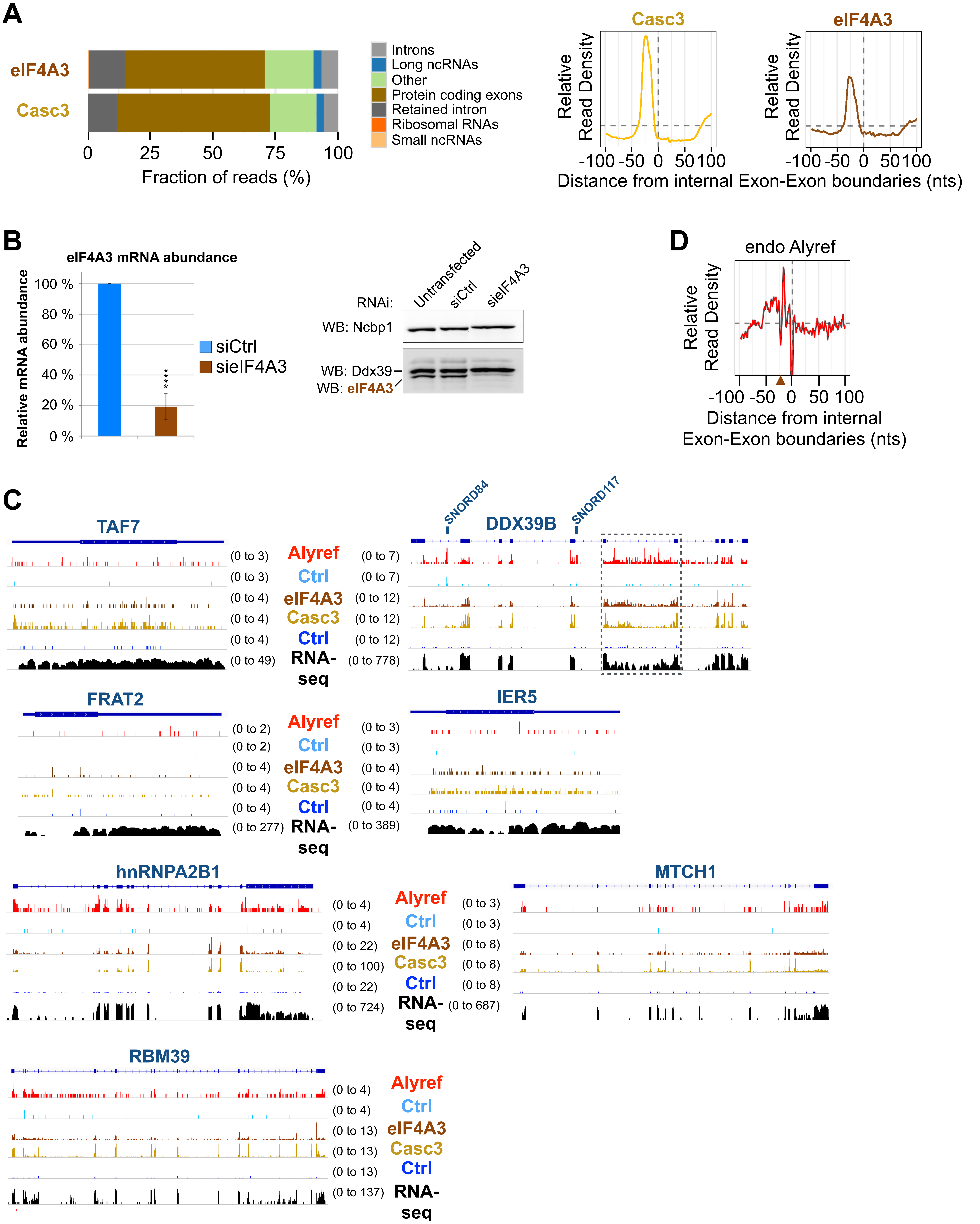

Figure S4. The EJC binds to a variety of RNA species and tethers Alyref. Related to Figure 5. A. Analysis of eIF4A3 and Casc3 iCLIP data from (Hauer et al. 2016). Distribution of reads and metagene analysis at internal exon-exon junctions. **B.** Knockdown efficiency of eIF4A3 analysed by RT-qPCR and Western blot. **C.** Additional iCLIP tracks of the transcripts studied in Figure 5. **D.** Analysis of endogenous Alyref iCLIP data from (Shi et al., 2017). Metagene analysis at internal exon-exon junctions. The EJC's position is inferred from Figure S4A and marked by a brown arrowhead.

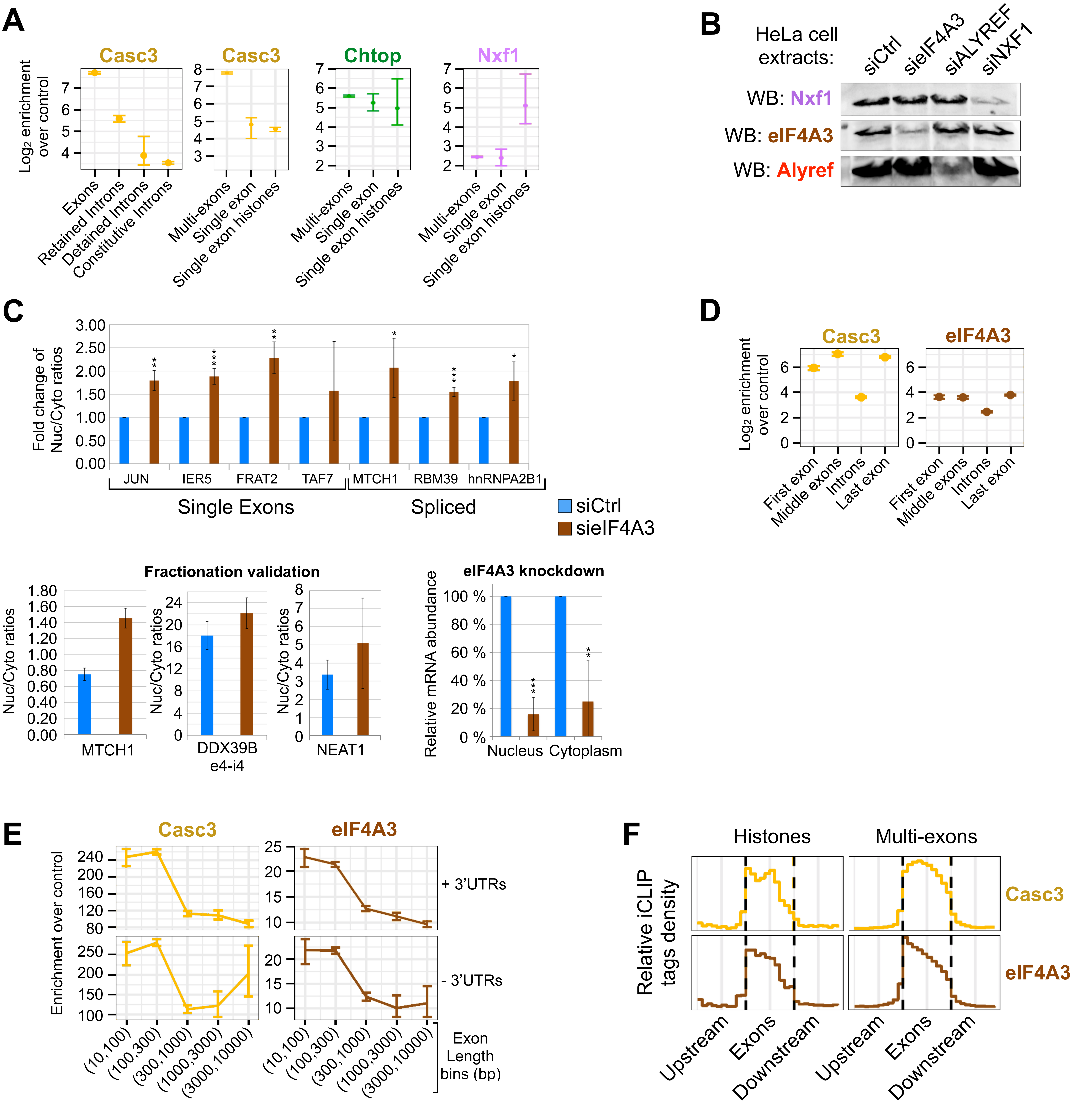

Figure S5. Impact of the EJC on RNA export. Related to Figure 5. A. Log2 enrichment of the indicated factors over their respective iCLIP negative control signals for the indicated genic regions and transcripts. **B.** Knockdown efficiency of RNA export factors in HeLa cells performed alongside the FISH experiment in Figure 5E. **C.** Fold change of Nuclear/ Cytoplasmic ratios upon eIF4A3 RNAi in 293T cells analysed by RT-qPCR. Validation of the fractionation was performed by analysing the Nuclear/Cytoplasmic ratios of an mRNA (MTCH1, 0.6 < ratio < 1.5), a pre-mRNA (DDX39B e4-i4, ratio >> 1), and a nuclear retained lncRNA (NEAT1, ratio >1). Means of three independent experiments +/- SD. *P< 0.05, **P< 0.01, ***P < 0.001 (t-test). **D.** Log2 enrichments of the indicated factors over their respective iCLIP negative control signals for the indicated genic regions. **E.** Binding enrichment of EJC core factors over Ctrl iCLIP as a function of increasing exon length with 3' UTRs included or not in the analysis. **F.** Deposition patterns of EJC core factors over single exon histones transcripts as in Figure 4C.

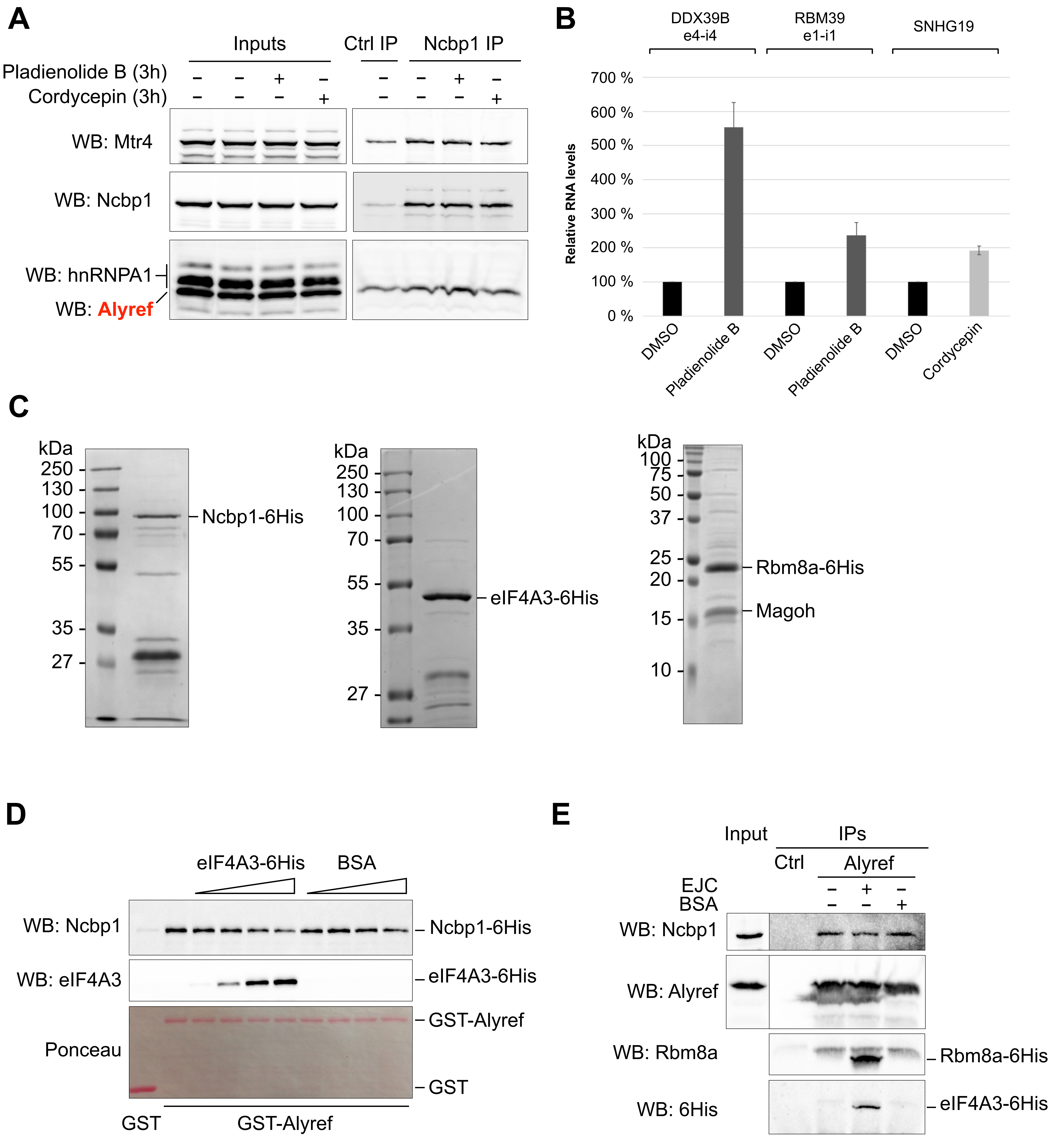

Figure S6. *In vitro* analysis of CBC, EJC, and Alyref interactions. Related to Figure 6. A. Western blot analysis of the co-immunoprecipitation of Alyref with the CBC subunit Ncbp1 from cells treated with the splicing inhibitor pladienolide B or the polyadenylation inhibitor cordycepin. hnRNPA1 is a negative control for interactions with TREX components and shows that RNase A treatment prevented co-IP of general mRNP binding proteins. **B.** The levels of the indicated transcripts were assessed by RT-qPCR in total RNA extracted from 10 % of the cells harvested for IP in Figure S6A. Values are mean +/- SD from two technical replicates. **C.** Recombinant proteins used in Figures S6D and S6F. **D.** GST or GST-Alyref pulldowns using recombinant Ncbp1-6His and increasing concentrations of recombinant eIF4A3-6His or BSA as competitors. **E.** Effect of recombinant EJC or BSA on Ncbp1 co-IP with Alyref.

Oligos pairs name	Forward primer sequence (5' - 3')	Reverse primer sequence (5' - 3')
JUN	GAACTGCACAGCCAGAACAC	TGGGTTGAAGTTGCTGAGG
IER5	TGGCTAACCTCATCAGCATC	GGGTTCATGTCTCTCAGCAC
TAF7	GCCTCTQCTGTGQGQQGGGC	CACGGTCCACTCTGACGATT
FRAT2	AGAAATGTATGCGCCAGGGT	TCGATGCAAGTAGCTGCCAT
DDX39B	CATGCGTCGGGATGTCCAGGAAA	GTCAAAGAGCTTCCGGTTCTTCTCG
DDX39B e6-i6	CATGCGTCGGGATGTCCAGGAAA	CACTTGAATGACAAGGGAGTCTGAGG
DDX39B e4-i4	TGTGTCACACTCGGGAGTTG	ACACCCTCACTCTCAGGTCT
RBM39 e1-e2	CAGCAGCAGCAATCTCTTCC	GAGCCTCAAGCATTGCTTCA
RBM39 e1-i1	CAGCAGCAGCAATCTCTTCC	AATGGAGCCAAACCTCGAGA
ENO1 e3-i3	CTAGAGCTCCGGGACAATGA	ATCATGGGTCACAGCAGGTT
SRSF7 e3-i3	AGATGCTATGAGTGTGGCGA	AAACACTAGTTTCTGCCTTGCT
hnRNPA2B1 e1-e2	GAAATCGGGCTGAAGCGAC	CACACAGTCTGTAAGCTTTCCC
hnRNPA2B1 e1-i1	GAAATCGGGCTGAAGCGAC	ATTTCCTGCCTCTCTCCCAC
hnRNPA2B1	TGATGACCATGATCCTGTGGA	CCTAGAACTCTGAACTTCCTGC
MTCH1	GAGGCCAAGTACAGTGGTGT	GTCATCCACCAGGTAGGCAT
eIF4A3	TTGGATGTCCCTCAGGTGTC	TCTCTGAGGATGCGGATGTC
TARDBP e1-i1	CTGCTTCGGTGTCCCTGT	CAATGCAGAAGCCGAGGC
TARDBP e1-e2	CTGCTTCGGTGTCCCTGT	CAGCCGGACACCTCTCATAC
CHTOP e2-e3	CACCACCAAGATGTCTCTAAATGAGCG	CTGCTTAAGTTTTAATGCTGCCTGGAC
CHTOP e5-3UTR	GAATCGGTGGTAGAGGTCGG	GTCTCTCATGGGAGGATGGG
CHTOP e4-i4	ACGAGGCCTACCCATAATCC	CCATAAGCAGCCCAAGAGAAAAGG
CHTOP 1kb post pA	TGCTCACTACAGTTCCCAGG	TTGTGTTGGTATGGCAAGCC
CHTOP 3kb post pA	AGACCTGACTACCTGCCCTA	CCTCTGTGAGCTCCAAAGGA
CHTOP 5.5kb post pA	TGATGGATGCCAGGCAGTAA	TGCATCTCCATTCAGCAGGA
ACTB unspliced	TCAAGGTGGGTGTCTTTCCT	CCTGCTTGCTGATCCACATC
ACTB 1kb ppA	TGCCTTCCCTCTGCTAGAAG	TGTGCACAGTTGAGAGTCCA
ACTB 2kb ppA	CCAACCAGATGTGTTCCGTG	CAAGACCACCACCACAATCG
ACTB 3kb ppA	AGAGGAAGAGGGCCAGAAAC	TGCAGTGACACAATCTTGGC
NEAT1	GCTTGGAACCTTGCTTCAAG	GGTGGGTAGGTGAGAGGTCA
SNHG19	CGTCCAGGCCTGGCCTAC	GCTCGCGACGAAACCTGC

Table S1. DNA oligonucleotides used in this study. Related to STAR Methods.
